# Supplementary material for: A landscape of response to drug combinations in non-small cell lung cancer
Source: Nat Commun. 2023 Jun 28;14:3830. doi: 10.1038/s41467-023-39528-9 (PMC10307832; doi:10.1038/s41467-023-39528-9)
Supplement: Supplementary file 1 — Supplementary Information [file 41467_2023_39528_MOESM1_ESM.pdf]

Supplementary notes and supplementary figures for paper "A landscape of response to drug combinations in non-small cell lung cancer"

Nishanth Ulhas Nair[1], Patricia Greninger[2], Xiaohu Zhang[6], Adam A. Friedman[2], Arnaud Amzallag[2], Eliane Cortez[2], Avinash Das Sahu[3], Joo Sang Lee[4], Anahita Dastur[2], Regina K. Egan[2], Ellen Murchie[2], Michele Ceribelli[6], Giovanna S. Crowther[2], Erin Beck[6], Joseph McClanaghan[2], Carleen Klump-Thomas[6], Jessica L. Boisvert[2], Leah J. Damon[2], Kelli M. Wilson[6], Jeffrey Ho[2], Angela Tam[2], Crystal McKnight[6], Sam Michael[6], Zina Itkin[6], Mathew J. Garnett[5], Jeffrey A. Engelman[2], Daniel A. Haber[2,6], Craig J. Thomas[7,8,*], Eytan Ruppin[1,*], Cyril H. Benes[2,*]

1 – Cancer Data Science Laboratory, Center for Cancer Research, National Cancer Institute, National Institutes of Health, Bethesda, USA.

2 – Massachusetts General Hospital, Harvard Medical School, Boston, USA.

3 – University of New Mexico, Comprehensive Cancer Center, Albuquerque, New Mexico, USA.

4 – Samsung Medical Center, Sungkyunkwan University School of Medicine, Suwon 16419, Republic of Korea.

5 – Wellcome Trust Sanger Institute, Wellcome Trust Genome Campus, Cambridge, CB10 1SA, UK.

6. Howard Hughes Medical Institute, Bethesda, MD, USA.

7. Division of Preclinical Innovation, National Center for Advancing Translational Sciences, National Institute of Health, Rockville MD 20850, USA.

8. Lymphoid Malignancies Branch, Center for Cancer Research, National Cancer Institute, National Institutes of Health, Bethesda, MD 20892, USA.

* –These authors jointly supervised this work. Cyril H. Benes – cyrilbenes@gmail.com; Eytan Ruppin – eytan.ruppin@nih.gov; Craig J. Thomas – craigt@mail.nih.gov

Supplementary Notes

1. Synergy computation

Synergy scores are computed using the Bliss model[1]. The lower the score, the more synergistic the drug-combination is. A drug combination is synergistic if its score is less than 1.

We defined a drug pair to be synergistic in the following manner: In each cell line and combination (for a particular library drug dose), we have cell counts measurements (day 6) upon library+anchor (LA) combination, library-only treatment (L), anchor-only treatment (A) and with only DMSO control treatment (C). Synergy score = (LA/A) / (L/C).

Consider the following example to calculate synergy shown in Supplementary Table 1:

Supplementary Table 1: Example to calculate synergy shown.

Cell Line	Anchor ID	Library ID	Cell Count (from drugged plates data)
X	DMSO	DMSO	500
X	DMSO	L	450
X	A	DMSO	400
X	A	L	150

Library drug alone response Plate (I) compared to DMSO control = 450 / 500

Library + Anchor Plate (II) Compared to Anchor alone = 150 / 400

Synergy (delta from bliss) = (Library + Anchor Plate (II) Compared to Anchor alone) / Library drug alone response Plate (I) compared to DMSO control = (150/400) / (450/500)

We calculate synergy for each cell line and for every drug pair in each cell line for all doses D1 to D5 (for library drugs). We then compute the median value of the 2 replicates to get a synergy value for each drug pair for each cell line for a particular library dose.

2. Coverage analysis

We ask the question: for a given anchor, what is the minimum number of library drugs required so that at least 80% of the cell lines have one high synergy?

To address the above question, we consider the 21x242 drug combos across 81 cell lines. High synergy was defined based on second-best synergy (top 5% threshold). For each anchor, we have a binary matrix for all cell lines and libraries – a matrix of 1s and 0s, where 1 stands for high synergy and 0 otherwise. We used a simple greedy algorithm to compute the minimum no. of library drugs so that at least 80% of the cell lines have one high synergy. Since it is a greedy algorithm, it is an approximate solution.

The greedy algorithm used is similar to the classic greedy algorithm for the set covering problem[2-4].

Our greedy algorithm is as follows:

a) For a given anchor, we have a binary matrix (say M) for all cell lines and libraries – a matrix of 1s and 0s, where 1 stands for high synergy and 0 otherwise. Let D be the counter for the no. of drugs considered. Set D=0. Also set the cell lines considered as PC = NULL SET.

b) We pick the library drug with the maximum high synergy cell lines for the given anchor in matrix M. Set D = D + 1.

c) We remove those high synergy cell lines from M (let C be the names of those cell lines). So, we get a new smaller matrix for M. Calculate PC = union(PC, C).

d) Check if (length_of_PC / total_no_of_cell_lines)*100 < 80%. If so, repeat from step b.

We repeated the above analysis for different percentages of cell lines (from 50% to 100%).

3. Potential mechanisms underlying some notable synergistic combinations

The first involves the NMPRT/NAMPT inhibitor daporinab (FK866), which was seen to yield HSA and synergies across many cell lines in combination with olaparib. PARP enzymes are thought to consume a large amount of cellular NAD as their substrate for PARylation on targets. Daporinab, by inhibiting NAMPT, lowers the levels of NAD in cells and this change in substrate availability has been previously suggested to underlie the synergy between PARP and NAMPT inhibitors[5] with potential impact for the treatment of Ewing's sarcoma[6].

Second, a number of synergies were observed with the insulin/insulin growth factor receptors inhibitor BMS754807. This outcome most likely corresponds to the well described feedback inhibition on IRS1(Insulin Receptor Substrate 1), a key adaptor in the Insulin Receptor pathway that is subject to inhibitory phosphorylation in an mTORC1 dependent manner and was recently described to imply degradation of IRS1 following phosphorylation by mTORC1 itself[7,8] BMS754807 synergizes less with BYL719 or GDC0941 than with OSI-027 even though mTORC1 is under the control of PI3K via AKT phosphorylation of TSC (Manning & Cantley, 2007). This is possibly due to the presence of other compensatory feedback loops that are not modulating the viability outcome when mTORC1 itself is targeted. In contrast to the PI3K inhibitors alpelisib (BYL719, PI3Kalpha) and pictilisib (GDC0941, pan PI3K), the MTORC inhibitor OSI027 synergizes broadly with the farnesyl transferase inhibitor tipifarnib (Figure 3G, Figures 6-7). This is an unexpected outcome that seems to indicate either unappreciated activity of farnesyl transferase linked to mTORC1/2, perhaps linking HRAS to mTORC or an unsuspected off target of one of the two inhibitors. Interestingly, a recent study identified MTORC2 as a direct binding partner of RAS proteins albeit not specific to HRAS over other RAS isoforms[9].

Thirdly, targeting the RB pathway has been a long-standing interest in cancer therapeutics[10]. Inhibition of CDK4/6, which regulates cell cycle entry via the control of RB1/E2F complex, has in recent years been employed in multiple clinical settings. Single agent activity has been observed

in preclinical models across different cancer types and promising results obtained in non-small-cell lung cancer (NSCLC) models[11]. In addition to improving the outcome of hormonal therapy in ER positive breast cancer inhibitors[12] of CDK4/6 are considered candidate combination agents in several cancers[13]. CDK4 depletion in a mouse model of NSCLC driven by KRAS induces senescence and tumor regression[14] and single agent activity of abemaciclib (LY2835219) a potent CDK4/6 inhibitor was seen in KRAS driven NSCLC human tumors[15]. However, further exploration of this paradigm has not led to improvement in overall survival of patients in the single agent setting[16]. More recently, a preclinical study reported activity of CDK4/6 targeting in NSCLC with loss of SMARCA4 (a component of the SWI/SNF chromatin regulator complex)[17]. There is thus considerable interest in building combinatorial strategies around CDK4/6 inhibition, in particular for KRAS mutant NSCLC. Here we identify mTOR and MEK inhibitors as best combination partners for palbociclib (Supplementary Figure 3). However, overall, our results in vitro and with limited time of exposure to drugs, show a modest number of synergies with high sparsity across cell lines. Nevertheless, longer time of treatment or non-cell autonomous effects could make these combinations beneficial in patients. Several clinical trials are poised to test the efficacy of MAPK pathway inhibitors in combination with CDK4/6 inhibition in NSCLC (for example, NCT03170206 for MAPK and NCT03065062 for mTOR/PI3K) and future combinations would be critical to address emerging resistance mechanisms[18]. In addition, different CDK4/6 inhibitors vary in their target selectivity such that across the three approved CDK4/6 inhibitors, specific combinations might display differential synergies and/or clinical benefit[19].

Our fourth set of interesting combinations involves SRC kinases. These enzymes, including p60c-Src and the closely related Src Family Kinases (SFK) members FYN and YES, are a group of non-receptor tyrosine kinases that have long been implicated in several hallmarks of cancer[20]. In particular, SFKs regulate adhesion and motility mediated by integrins in coordination with the tyrosine kinase FAK as well as growth factor signaling. SFK are for example known to play a role in the activation of the MEK-ERK/MAPK pathway in several contexts and recent mechanistic studies support a model whereby SFKs can coordinate various signaling inputs with growth factor sensing by RTKs[21]. Their targeting in tumors has long been elusive however and somewhat surprisingly, no mutations are found in these kinases in tumors. The multi-targeted kinase inhibitor dasatinib is a potent inhibitor of Bcr-Abl and was approved for use in Philadelphia positive chronic

myelogenous leukemia (CML) and Acute Lymphoblastic Leukemia (Ph+ ALL) in 2006[22]. It also potently inhibits SRC and other SFKs and was used here to probe the potential of an approved drug targeting SFKs to yield beneficial combinations in NSCLC. We found that TAE-684 synergizes broadly with dasatinib (Supplementary Figure 3,5). This is surprising since the kinase inhibitor TAE-684 was designed to inhibit the ALK tyrosine kinase which is an oncogene in NSCLC when fused to EML4[23]. ALK is not normally expressed in NSCLC and ALK inhibitors are thus not broadly expected to be active outside of the ALK fusion driver context. This synergistic interaction between dasatinib and TAE-684 is thus most likely due to targeting of other kinase(s) than ALK, possibly IGF1R[24]. Two Insulin Receptor (IR) / Insulin Growth Factor 1 Receptor (IGF1R) inhibitors were used. Intriguingly, only one, BMS-754807 but not linsitinib displayed a very high number of synergies with dasatinib and indeed was the top synergizing drug with dasatinib. This strongly suggests that the synergies seen with the BMS compound are either not due to IR/IGF1R targeting or that additional targeting is needed to yield synergy (Supplementary Figure 4,5). This illustrates again the challenge brought by polypharmacology when trying to assign mechanisms to the observed synergies as well as how polypharmacology can result in differential synergistic outcome across compounds that are as single agents yielding similar activity profiles.

Fifth, a number of strong synergies were seen with the pan Aurora Kinase (AURK) inhibitor tozasertib (VX-680, used as an anchor) (Figure 3G, 4D, Supplementary Figures 7-8). Three additional AURK inhibitors were used in the drug library: Barasertib (AZD-1152) which is selective for Aurora Kinase B over Aurora Kinase A, alisertib (MLN8237) and ENMD-981693 which are selective for Aurora kinase A. Synergistic activity was detected between AURK inhibitors and HDAC inhibitors (Supplementary Figure 7) in line with previous reporting[25]. Interestingly, while both AURK and Cyclin Dependent Kinases are involved in cell cycle progression CDK inhibitors did not display the same broad pattern of synergies as AURK inhibitors (Supplementary Figure 7). Thus, cell cycle inhibition does not appear to be sufficient to explain the AURK inhibitors results and perhaps a more specific outcome of inhibiting mitotic progression with AURK inhibitors is at play or other non-mitotic targets[26-28] of AURK are involved. Across anchors, different AURK inhibitors display differential synergy profiles. For example, while both barasertib and alisertib synergize with the BCL2 family targeting compound

navitoclax, ENMD-981693 shows more synergies across cell lines with the CHK inhibitor AZD7762 than other AURK inhibitors. In contrast, all 4 AURK inhibitors tested synergize with the HDAC inhibitor vorinostat across many cell lines, albeit with only partial overlap of cell lines presenting synergy (Supplementary Figure 7). Thus, inhibition of either AURKA, AURKB (and AURKC) appears to have distinct outcomes in the combination setting. The mechanistic underlying of this observation is unclear but could be either a specific, possibly non-mitotic function of AURKs, a specific state of cell cycle arrest obtained with one inhibitor versus another, or alternate targets engaged by different inhibitors. Nevertheless, here and in other combination screens[29] targeting AURK in combination with different growth factors, survival and DNA damaging agents could yield relatively frequent synergistic outcomes. Interestingly, AURKA has been linked to PI3K inhibition response in breast cancer and to resistance to EGFR inhibition in NSCLC[30,31].

Sixth, the BCL2 family inhibitor synergizes with many drugs in the present study in keeping with previous results obtained in our studies on melanoma cell lines where navitoclax was characterized as a broad sensitizer to many other drugs[32]. This makes intuitive sense for a pro-apoptotic agent. In particular, some but not all inhibitors of the cell cycle present with numerous synergies when combined with navitoclax. For example, dinaciclib an inhibitor of multiple CDKs, BI-2536 and GW843682X inhibitors of Polo Like Kinases (PLK), or AT9283, alisertib and barasertib, inhibitors of AURKs are some of the drugs with the most synergies. There is precedent for the combination of dinaciclib and ABT263 to synergize via downregulation of MCL1, a major resistance factor to navitoclax as mentioned in the results section. Somewhat less expected are synergies observed between the ETC complex V inhibitor oligomycin and navitoclax, although a recent report on CLL identified oxidative phosphorylation as a regulator or BCL2 inhibitor sensitivity and demonstrated synergy between oligomycin and BCL2 targeting in lymphoid cells[33]. BCL2 family members are not only involved in apoptosis by regulating the release of cytochrome C from the mitochondria but also in the maintenance of mitochondrial network function through regulation of fission-fusion cycle and possibly other means[34]. Thus, the observed synergies between complex V inhibition and BCL2 inhibition could represent impact on mitochondrial integrity or sensitization to mitochondrial release of cytochrome C.

Seventh, the mitochondrial ATPsynthase inhibitor oligomycin was ranked 6th when considering median HSA score across cell lines for trametinib. This represented strong but relatively uncommon synergies and thus was also not flagged in the impact score analysis. Notably, oligomycin was the only drug with no obvious signaling network connection to MEK in the top 12 drugs ranked by median HSA. Oligomycin was also synergistic with OSI-27, pictilisib, dasatinib, navitoclax and perhaps less surprisingly with phenformin. These results suggest that disruption of ETC, while not necessarily lethal on its own, is creating vulnerability to core growth factor signaling inhibition by PI3K or ERK pathway inhibition.

4. Self-addition breaking and limitation of statistical independence modeling

A simple explanation for self-additive coherence breaking is as follows: Consider a scenario where doubling the dose of a drug yields a doubling of the viability effect. Then consider what happens if the outcome of dose A for this drug is 80% viability. Doubling the dose (adding the same treatment to itself, Ax2), based on statistical independence would be expected to yield 64% viability (0.8x0.8). Thus, in this case yielding a synergy score of 0.625 (0.08/0.64). Now consider the same drug but with an initial dose yielding 40% viability. In this case the expected outcome is 16% (0.4x0.4; or 40% of 40%) and the observed outcome is 20%, meaning that no synergy is detected. Now, consider a drug that has a shallower dose response curve with a doubling of the dose yielding a 1.2-fold change in viability. This time, starting at 80% for dose A the expected outcome of 64% for (A)x2 is compared to an observed outcome of 0.67 (0.8/1.2) which does not represent synergy based on statistical independence (67% compared to 64%). Thus, both steepness of the curve and dosing (where on the dose response curve the data is acquired) impact the synergy call outcome. Importantly, analysis of single agent data in the present screen reveals that dosing yielded a broad range of viability values (Supplementary Figure 1) and that viability ratio for two consecutive doses chosen experimentally to be $\sqrt{10}$ apart (every other dose matches a 10-fold dilution) has a median value of 1.04 (1.14 for the top 25% ratio values). By contrast, the HDAC inhibitor vorinostat that shows self-additive synergy has a median ratio of viability across two consecutive doses of 1.44 (2.11 for the top 25% ratio values).

5. Comparison of *in vitro* and *in vivo* NSCLC models

Gao et al. (2015)[35] tested 5 drug combinations across 36 NSCLC mouse PDX models. While none of these drug combinations were exactly the same as what we used in our *in vitro* original drug combination screens, 3 drug combinations had similar drug targets. These three drug combinations in the Gao paper are: BKM120 (panPI3K inhibitor) and binimetinib (MEK inhibitor); BYL719 (PI3Kalpha inhibitor) and LGH447 (PIM inhibitor); LFW527 (IGF1R inhibitor) and binimetinib (MEK inhibitor).

Since, there is no clear concept of synergy in this mouse models dataset, in the Gao et al paper[35], we checked the percentage of PDX models in which drug combinations are more effective than both individual drugs (based on the percent tumor volume change after treatment; one-sided Wilcoxon rank-sum test, $P < 0.05$). We compared it with the percentage of highly synergistic cell lines in the correspondingly mapped drug combinations in our original *in vitro* screen (top 5 percentile chosen as the threshold of high synergy). The results are summarized in Supplementary Table 2. As seen, we see a very good concordance between our drug combination in vitro screen and in the PDX models based on the fraction of highly synergistic/effective models for PI3K and MEK inhibitors, PI3K and PIM inhibitors, IGF1R and MEK inhibitors.

Supplementary Table 2: Comparing drug combinations between *in vitro* and *in vivo* NSCLC models.

**Drug targets**	**PDX screen drug combination (percentage of NSCLC PDX models in which the combinations is more effective than both individual drugs is shown).**	**Original *in vitro* screen drug combination for the mapped drugs based on similar targets (percentage of highly synergistic cell lines shown)**
PI3K & MEK inhibitor combination	BKM120 & Binimetinib = **20.69%** (6 out of 29 models)	Alpelisib & Trametinib = 24.32%<br><br>Alpelisib & Selumetinib =16.22%<br><br>Pictilisib & Trametinib = 31.58%<br><br>Pictilisib & Selumetinib = 22.37%<br><br>Mean value of high synergies of these combinations = **23.62%**
PI3K & PIM inhibitor combination	BYL719 & LGH447 = **10.34%** (3 out of 29 models)	Alpelisib & AZD1208 = 8.11%<br><br>Alpelisib & SGI-1776 = 4.05%<br><br>Pictilisib & AZD1208 = 17.11%<br><br>Pictilisib & SGI-1776 = 3.95%<br><br>Mean value of high synergies of these combinations = **8.31%**
IGF1R & MEK inhibitor combination	LFW527 & Binimetinib = **12% (**3 out of 25 models).	Linsitinib & Trametinib = 10.96%<br><br>Linsitinib & Selumetinib = 9.59%<br><br>Mean value of high synergies of these combinations = **10.28%**

Supplementary Table 3: Cell lines used in this study.

ID	Cell Line	Source	CatalogNumber
850	201T	UPMC	NA
752	A-427	ATCC	HTB-53
677	A549	ATCC	CCL-185
888	ABC-1	JHSF	JCRB0815
756	BEN	DSMZ	ACC 254
851	CAL-12T	DSMZ	ACC 443
679	ChaGo-K-1	ATCC	HTB-168
864	COR-L105	ECACC	92031918
865	COR-L23	ECACC	92031919
886	EBC-1	JHSF	JCRB0820
854	EPLC-272H	DSMZ	ACC 383
890	H3255	NCI	NA
872	HARA	JHSF	JCRB1080.0
855	HCC-15	DSMZ	ACC 496
856	HCC-366	DSMZ	ACC 492
857	HCC-44	DSMZ	ACC 534
858	HCC-78	DSMZ	ACC 563
859	HCC-827	DSMZ	ACC 566
860	LCLC-103H	DSMZ	ACC 384
861	LCLC-97TM1	DSMZ	ACC 388
871	LK-2	JHSF	JCRB0829
862	LOU-NH91	DSMZ	ACC 393
879	LU99A	JHSF	JCRB0044
801	NCI-H1299	ATCC	CRL-5803
1243	NCI-H1395	ATCC	CRL-5868
812	NCI-H1437	ATCC	CRL-5872
813	NCI-H1563	ATCC	CRL-5875
815	NCI-H1623	ATCC	CRL-5881
1245	NCI-H1648	ATCC	CRL-5882
791	NCI-H1650	ATCC	CRL-5883
816	NCI-H1651	ATCC	CRL-5884
792	NCI-H1666	ATCC	CRL-5885
818	NCI-H1703	ATCC	CRL-5889
819	NCI-H1734	ATCC	CRL-5891
820	NCI-H1755	ATCC	CRL-5892
794	NCI-H1792	ATCC	CRL-5895
821	NCI-H1793	ATCC	CRL-5896
823	NCI-H1915	ATCC	CRL-5904
824	NCI-H1944	ATCC	CRL-5907
755	NCI-H1975	ATCC	CRL-5908
1136	NCI-H1993	ATCC	CRL-5909
796	NCI-H2009	ATCC	CRL-5911
825	NCI-H2023	ATCC	CRL-5912
827	NCI-H2085	ATCC	CRL-5921
847	NCI-H2087	ATCC	CRL-5922
841	NCI-H2170	ATCC	CRL-5928
832	NCI-H2228	ATCC	CRL-5935
800	NCI-H23	ATCC	CRL-5800
833	NCI-H2342	ATCC	CRL-5941
834	NCI-H2347	ATCC	CRL-5942
835	NCI-H2405	ATCC	CRL-5944
1180	NCI-H3122	DFCI	NA
802	NCI-H358	ATCC	CRL-5807
840	NCI-H441	ATCC	HTB-174
753	NCI-H460	ATCC	HTB-177
842	NCI-H520	ATCC	HTB-182
803	NCI-H522	ATCC	CRL-5810
797	NCI-H596	ATCC	HTB-178
806	NCI-H647	ATCC	CRL-5834
807	NCI-H650	ATCC	CRL-5835
799	NCI-H661	ATCC	HTB-183
680	NCI-H727	ATCC	CRL-5815
808	NCI-H838	ATCC	CRL-5844
868	PC-14	ECACC	90071810
877	PC-3 [JPC-3]	JHSF	JCRB0077
870	RERF-LC-KJ	JHSF	JCRB0137
848	SK-LU-1	ECACC	93120835
843	SK-MES-1	ATCC	HTB-58
844	SW1573	ATCC	CRL-2170
839	SW900	ATCC	HTB-59
678	UMC-11	ATCC	CRL-5975
8018	Calu-6	ATCC	HTB-56
8040	EKVX	NCI	NA
8072	HOP-62	NCI	NA
8075	IA-LM	RIKEN	RCB0554
8103	LC-2-ad	RIKEN	RCB0440
8111	LXF-289	DSMZ	ACC265
8130	NCI-H1355	ATCC	CRL-5865
8132	NCI-H2126	ATCC	CCL-256
8133	NCI-H322M	NCI	NA
8232	EMC-BAC-2	WTSI	NA

Supplementary Data

Supplementary data S1-S11 are provided separately in Excel files. Supplementary data S12 is a compressed (zip) folder.

Supplementary Figures

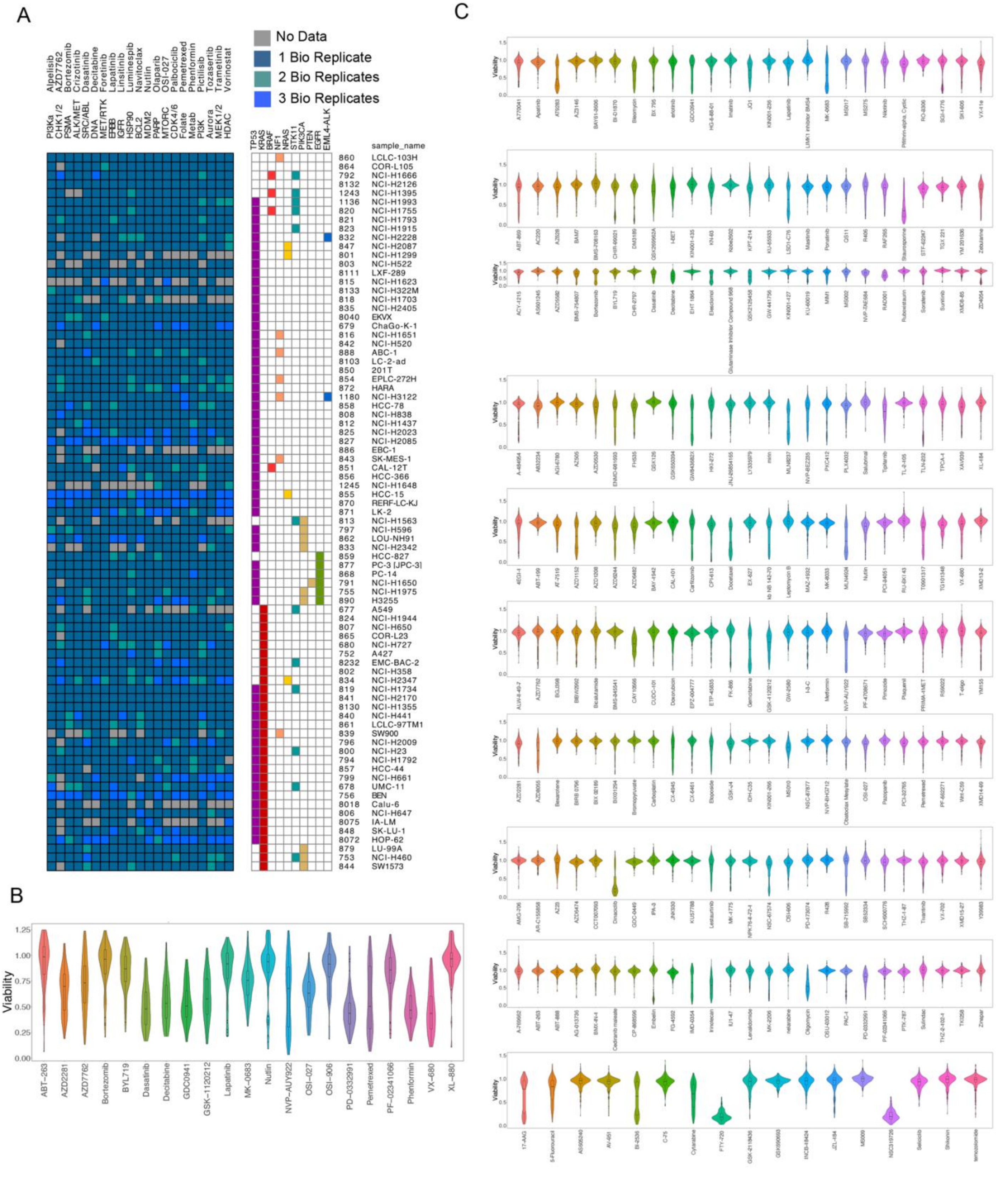
A
No Data
1 Bio Replicate
2 Bio Replicates
3 Bio Replicates
Alpelisib
AZD7762
Bortezomib
Crizotinib
Dasatinib
Decitabine
Foretinib
Lapatinib
Linsitinib
Luminespib
Navitoclax
Nutlin
Olaparib
OSI-027
Palbociclib
Pemetrexed
Phenformin
Pictilisib
Tozasertib
Trametinib
Vorinostat
PI3Ka
CHK1/2
PSMA
ALK/MET
SRC/ABL
DNA
MET/RTK
ERBB
IGFR
HSP90
BCL2
MDM2
PARP
MTORC
CDK4/6
Folate
Metab
PI3K
Aurora
MEK1/2
HDAC
TP53
KRAS
BRAF
NF1
NRAS
STK11
PIK3CA
PTEN
EGFR
EML4-ALK
sample_name
860 LCLC-103H
864 COR-L105
792 NCI-H1666
8132 NCI-H2126
1243 NCI-H1395
1136 NCI-H1993
820 NCI-H1755
821 NCI-H1793
823 NCI-H1915
832 NCI-H2228
847 NCI-H2087
801 NCI-H1299
803 NCI-H522
8111 LXF-289
815 NCI-H1623
8133 NCI-H322M
818 NCI-H1703
835 NCI-H2405
8040 EKVX
879 ChaGo-K-1
816 NCI-H1651
842 NCI-H520
888 ABC-1
8103 LC-2-ad
850 201T
854 EPLC-272H
872 HARA
1180 NCI-H3122
858 HCC-78
808 NCI-H838
812 NCI-H1437
825 NCI-H2023
827 NCI-H2085
886 EBC-1
843 SK-MES-1
851 CAL-12T
856 HCC-366
1245 NCI-H1648
855 HCC-15
870 RERF-LC-KJ
871 LK-2
813 NCI-H1563
797 NCI-H596
862 LOU-NH91
833 NCI-H2342
859 HCC-827
877 PC-3 [JPC-3]
868 PC-14
791 NCI-H1650
755 NCI-H1975
890 H3255
677 A549
824 NCI-H1944
807 NCI-H650
865 COR-L23
680 NCI-H727
752 A427
8232 EMC-BAC-2
802 NCI-H358
834 NCI-H2347
819 NCI-H1734
841 NCI-H2170
8130 NCI-H1355
840 NCI-H441
861 LCLC-97TM1
839 SW900
796 NCI-H2009
800 NCI-H23
794 NCI-H1792
857 HCC-44
799 NCI-H661
678 UMC-11
756 BEN
8018 Calu-6
806 NCI-H647
8075 IA-LM
848 SK-LU-1
8072 HOP-62
879 LU-99A
753 NCI-H460
844 SW1573
B
Viability
1.25
1.00
0.75
0.50
0.25
0.00
ABT-263
AZD2281
AZD7762
Bortezomib
BYL719
Dasatinib
Decitabine
GDC0941
GSK-1120212
Lapatinib
MK-0683
Nutlin
NVP-AUY922
OSI-027
OSI-906
PD-0332991
Pemetrexed
PF-02341066
Phenformin
VX-680
XL-880
C
Viability

Supplementary Figure 1: Overview of the dataset and response to single agents. A: Coverage of the cell line collection. The 81 cell lines (rows) are shown with key cancer genes altered to the right of the heatmap displaying data acquired and passing quality control. The number of biological replicates (Bio Replicates) corresponding to independent days of cell seeding are indicated by the color scheme indicated at the top right. B. Viability response to anchor drugs, the distribution of viability values (across all 81 cell lines) is represented. C. Viability response to library drugs: For each library drugs the range of viability obtained with the indicated drug across the 81 cell line collection is plotted. All 5 doses are plotted together for each drug to demonstrate the overall range of viability obtained. The box plots have median center, 25 and 75 percentiles (Q1 and Q3) as bounds of the box, the minima and maxima being Q1 – 1.5xIQR and Q3 + 1.5xIQR (with IQR being interquartile range).

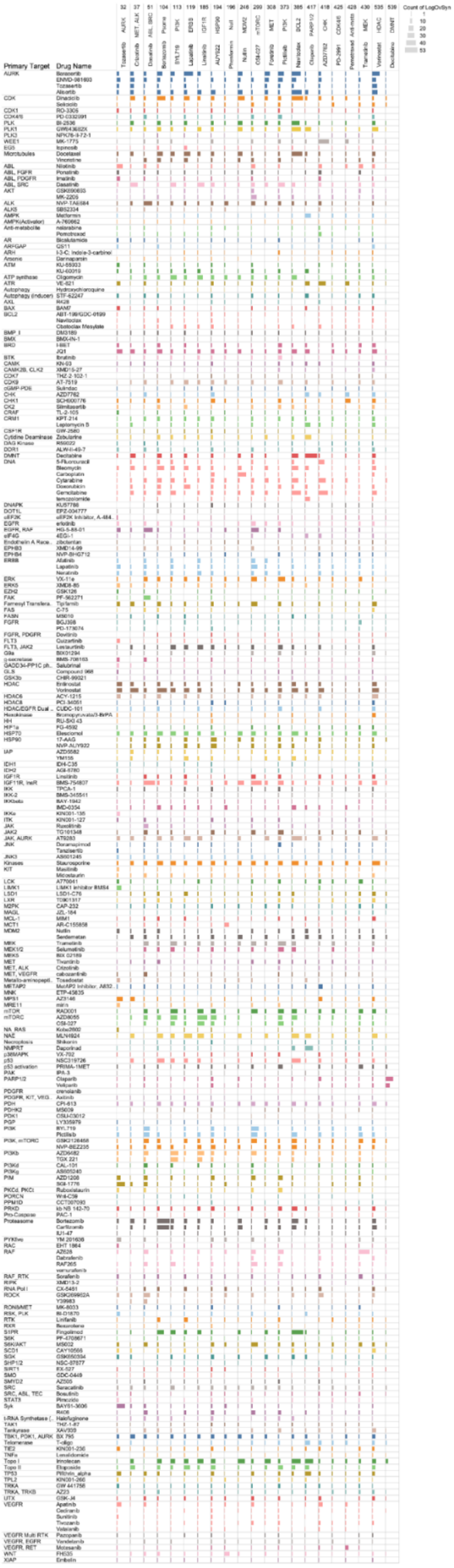

Supplementary Figure 2: Profile of synergy counts across all tested combinations. For each combination between the indicated library drug (row) and anchor drug (column) the number of cell lines harboring synergy is depicted by a bar. The size of the bar is proportional to the number of cell lines harboring synergy with maximum size set at 53 (maximum number of cell lines presenting with synergy). Synergy count is based on a synergy score threshold of 0.8.

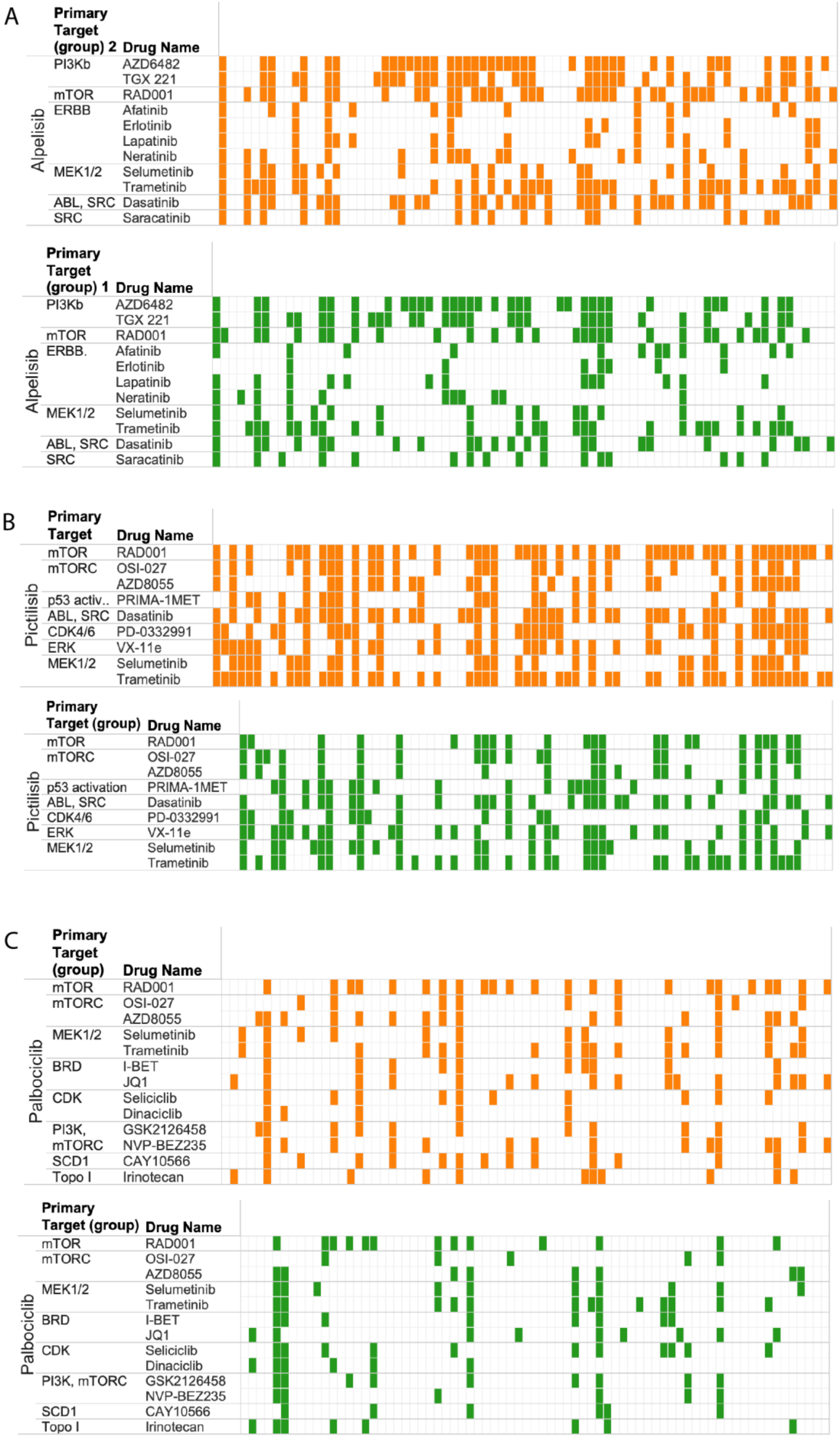
A
Primary Target (group) 2
Drug Name
Alpelisib
PI3Kb AZD6482
TGX 221
mTOR RAD001
ERBB Afatinib
Erlotinib
Lapatinib
Neratinib
MEK1/2 Selumetinib
Trametinib
ABL, SRC Dasatinib
SRC Saracatinib
Primary Target (group) 1
Drug Name
Alpelisib
PI3Kb AZD6482
TGX 221
mTOR RAD001
ERBB. Afatinib
Erlotinib
Lapatinib
Neratinib
MEK1/2 Selumetinib
Trametinib
ABL, SRC Dasatinib
SRC Saracatinib
B
Primary Target
Drug Name
Pictilisib
mTOR RAD001
mTORC OSI-027
AZD8055
p53 activ.. PRIMA-1MET
ABL, SRC Dasatinib
CDK4/6 PD-0332991
ERK VX-11e
MEK1/2 Selumetinib
Trametinib
Primary Target (group)
Drug Name
Pictilisib
mTOR RAD001
mTORC OSI-027
AZD8055
p53 activation PRIMA-1MET
ABL, SRC Dasatinib
CDK4/6 PD-0332991
ERK VX-11e
MEK1/2 Selumetinib
Trametinib
C
Primary Target (group)
Drug Name
Palbociclib
mTOR RAD001
mTORC OSI-027
AZD8055
MEK1/2 Selumetinib
Trametinib
BRD I-BET
JQ1
CDK Seliciclib
Dinaciclib
PI3K, mTORC GSK2126458
NVP-BEZ235
SCD1 CAY10566
Topo I Irinotecan
Primary Target (group)
Drug Name
Palbociclib
mTOR RAD001
mTORC OSI-027
AZD8055
MEK1/2 Selumetinib
Trametinib
BRD I-BET
JQ1
CDK Seliciclib
Dinaciclib
PI3K, mTORC GSK2126458
NVP-BEZ235
SCD1 CAY10566
Topo I Irinotecan

D

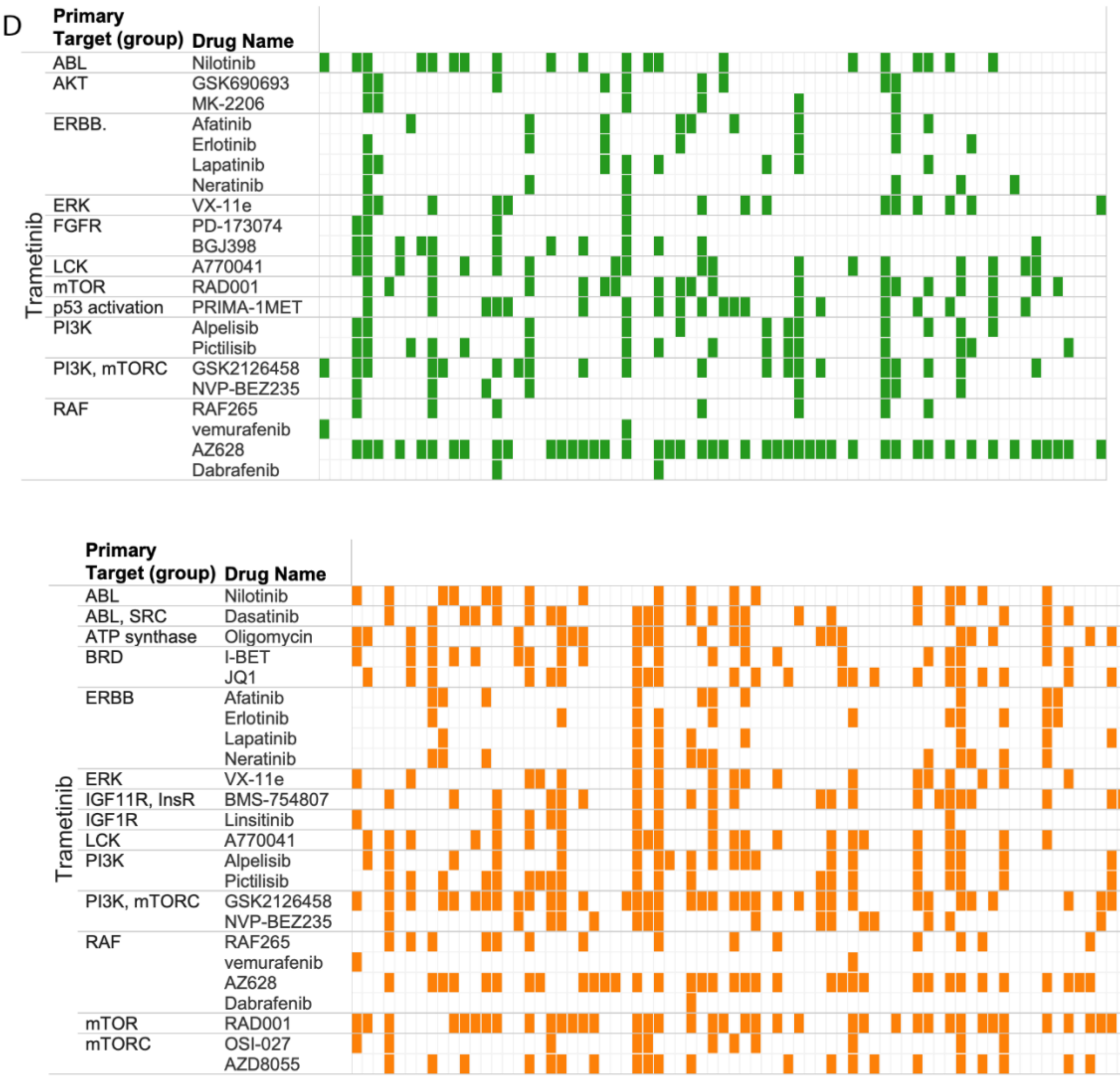
Primary Target (group)
Drug Name
Trametinib
ABL
Nilotinib
AKT
GSK690693
MK-2206
ERBB.
Afatinib
Erlotinib
Lapatinib
Neratinib
ERK
VX-11e
FGFR
PD-173074
BGJ398
LCK
A770041
mTOR
RAD001
p53 activation
PRIMA-1MET
PI3K
Alpelisib
Pictilisib
PI3K, mTORC
GSK2126458
NVP-BEZ235
RAF
RAF265
vemurafenib
AZ628
Dabrafenib
Primary Target (group)
Drug Name
Trametinib
ABL
Nilotinib
ABL, SRC
Dasatinib
ATP synthase
Oligomycin
BRD
I-BET
JQ1
ERBB
Afatinib
Erlotinib
Lapatinib
Neratinib
ERK
VX-11e
IGF11R, InsR
BMS-754807
IGF1R
Linsitinib
LCK
A770041
PI3K
Alpelisib
Pictilisib
PI3K, mTORC
GSK2126458
NVP-BEZ235
RAF
RAF265
vemurafenib
AZ628
Dabrafenib
mTOR
RAD001
mTORC
OSI-027
AZD8055

E

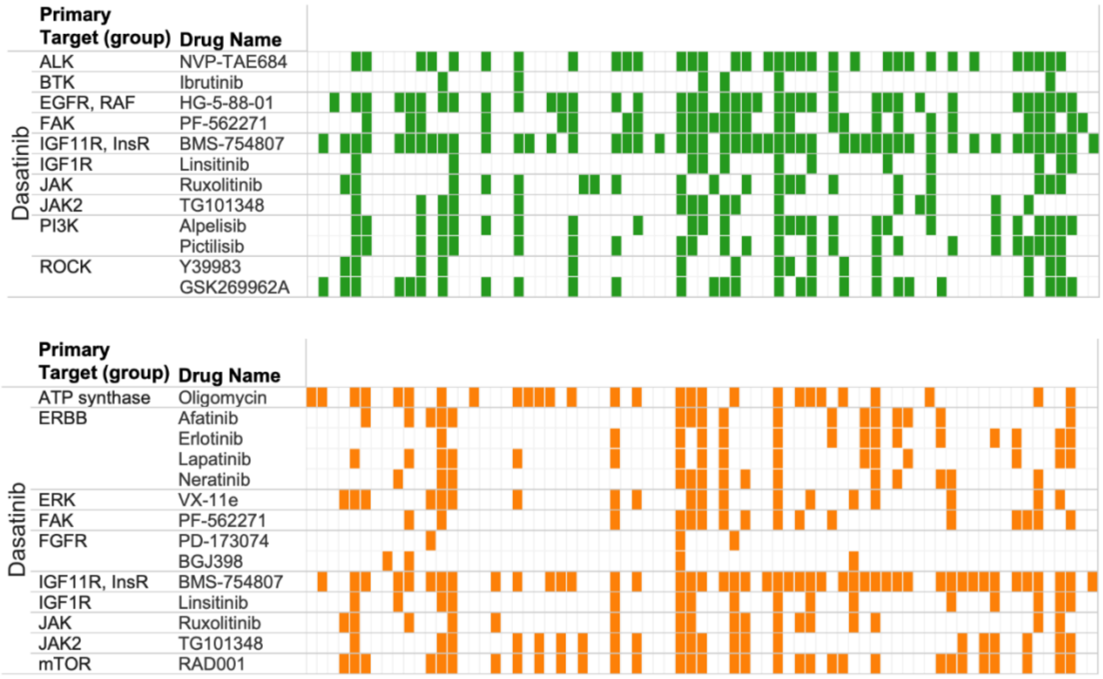
Primary Target (group)
Drug Name
Dasatinib
ALK
NVP-TAE684
BTK
Ibrutinib
EGFR, RAF
HG-5-88-01
FAK
PF-562271
IGF11R, InsR
BMS-754807
IGF1R
Linsitinib
JAK
Ruxolitinib
JAK2
TG101348
PI3K
Alpelisib
Pictilisib
ROCK
Y39983
GSK269962A
Primary Target (group)
Drug Name
Dasatinib
ATP synthase
Oligomycin
ERBB
Afatinib
Erlotinib
Lapatinib
Neratinib
ERK
VX-11e
FAK
PF-562271
FGFR
PD-173074
BGJ398
IGF11R, InsR
BMS-754807
IGF1R
Linsitinib
JAK
Ruxolitinib
JAK2
TG101348
mTOR
RAD001

Supplementary Figure 3: Pattern of synergies and HSA events across related drugs. Several examples of patterns obtained for drugs with related targets are shown for some of the anchors. A-E: Cell lines (columns) are in the same order across rows to allow for pattern comparison. Cell lines harboring HSA (orange) or synergy (green) are colored accordingly. The same thresholds for synergy and HSA are used across all panels. Anchors for the different panels are: A, Alpelisib (PI3Kalpha); B, Pictilisib (pan PI3K); C, Palbociclib (CDK4/6); D, Trametinib (MEK1/2); E, Dasatinib (ABL, SRC).

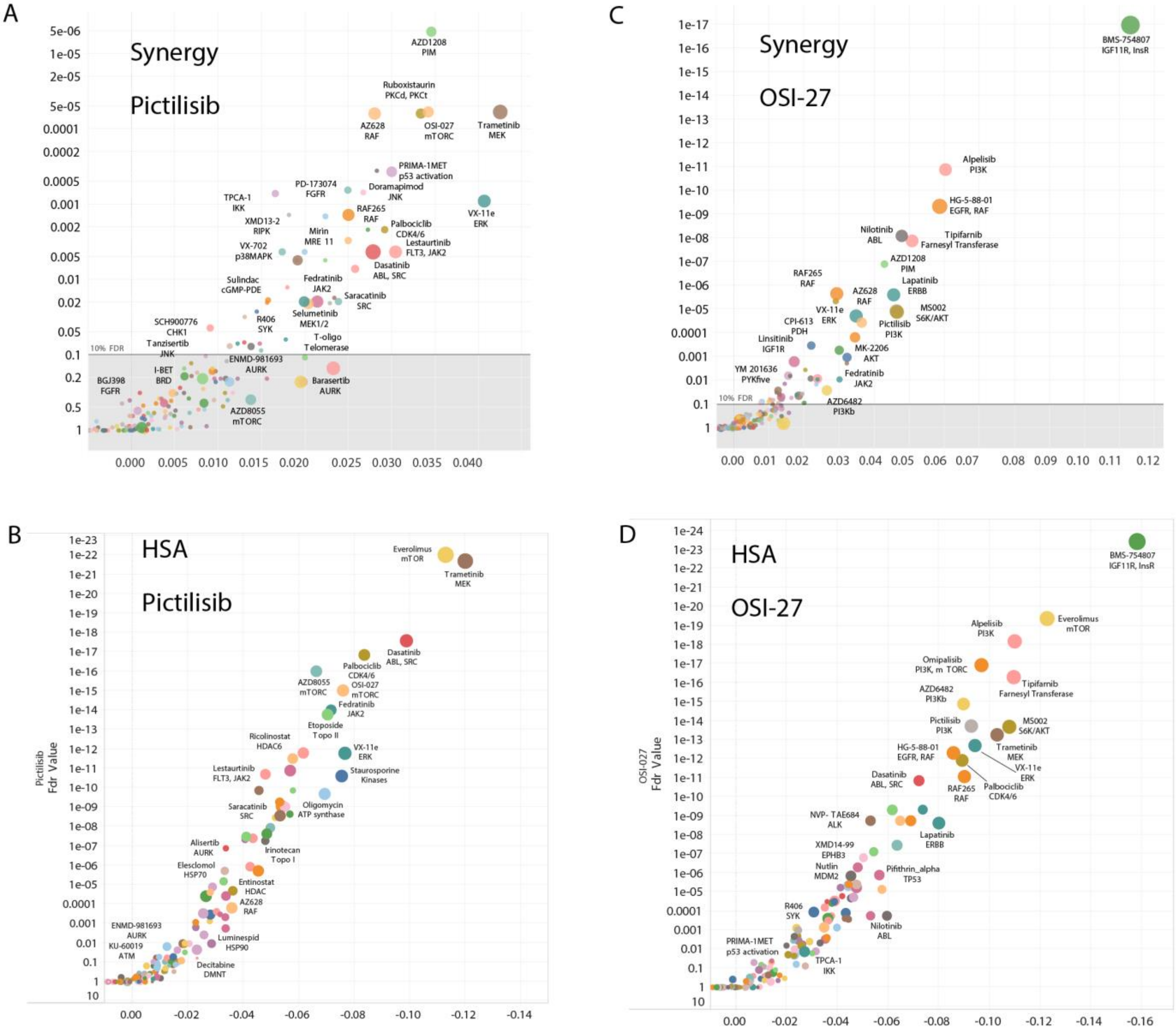
A
Synergy
Pictilisib
10% FDR
AZD1208
PIM
Ruboxistaurin
PKCd, PKCt
AZ628
RAF
OSI-027
mTORC
Trametinib
MEK
PRIMA-1MET
p53 activation
PD-173074
FGFR
Doramapimod
JNK
TPCA-1
IKK
VX-11e
ERK
RAF265
RAF
XMD13-2
RIPK
Palbociclib
CDK4/6
Mirin
MRE 11
Lestaurtinib
FLT3, JAK2
VX-702
p38MAPK
Dasatinib
ABL, SRC
Sulindac
cGMP-PDE
Fedratinib
JAK2
Saracatinib
SRC
Selumetinib
MEK1/2
R406
SYK
SCH900776
CHK1
T-oligo
Telomerase
Tanzisertib
JNK
ENMD-981693
AURK
I-BET
BRD
BGJ398
FGFR
Barasertib
AURK
AZD8055
mTORC
B
HSA
Pictilisib
Pictilisib
Fdr Value
Everolimus
mTOR
Trametinib
MEK
Dasatinib
ABL, SRC
Palbociclib
CDK4/6
AZD8055
mTORC
OSI-027
mTORC
Fedratinib
JAK2
Etoposide
Topo II
Ricolinostat
HDAC6
VX-11e
ERK
Lestaurtinib
FLT3, JAK2
Staurosporine
Kinases
Saracatinib
SRC
Oligomycin
ATP synthase
Alisertib
AURK
Irinotecan
Topo I
Elesclomol
HSP70
Entinostat
HDAC
AZ628
RAF
ENMD-981693
AURK
Luminespid
HSP90
KU-60019
ATM
Decitabine
DMNT
C
Synergy
OSI-27
10% FDR
BMS-754807
IGF11R, InsR
Alpelisib
PI3K
HG-5-88-01
EGFR, RAF
Nilotinib
ABL
Tipifarnib
Farnesyl Transferase
AZD1208
PIM
RAF265
RAF
Lapatinib
ERBB
AZ628
RAF
VX-11e
ERK
MS002
S6K/AKT
Pictilisib
PI3K
CPI-613
PDH
Linsitinib
IGF1R
MK-2206
AKT
YM 201636
PYKfive
Fedratinib
JAK2
AZD6482
PI3Kb
D
HSA
OSI-27
OSI-027
Fdr Value
BMS-754807
IGF11R, InsR
Everolimus
mTOR
Alpelisib
PI3K
Omipalisib
PI3K, m TORC
Tipifarnib
Farnesyl Transferase
AZD6482
PI3Kb
Pictilisib
PI3K
MS002
S6K/AKT
HG-5-88-01
EGFR, RAF
Trametinib
MEK
VX-11e
ERK
Dasatinib
ABL, SRC
Palbociclib
CDK4/6
RAF265
RAF
NVP- TAE684
ALK
Lapatinib
ERBB
XMD14-99
EPHB3
Nutlin
MDM2
Pifithrin_alpha
TP53
R406
SYK
Nilotinib
ABL
PRIMA-1MET
p53 activation
TPCA-1
IKK

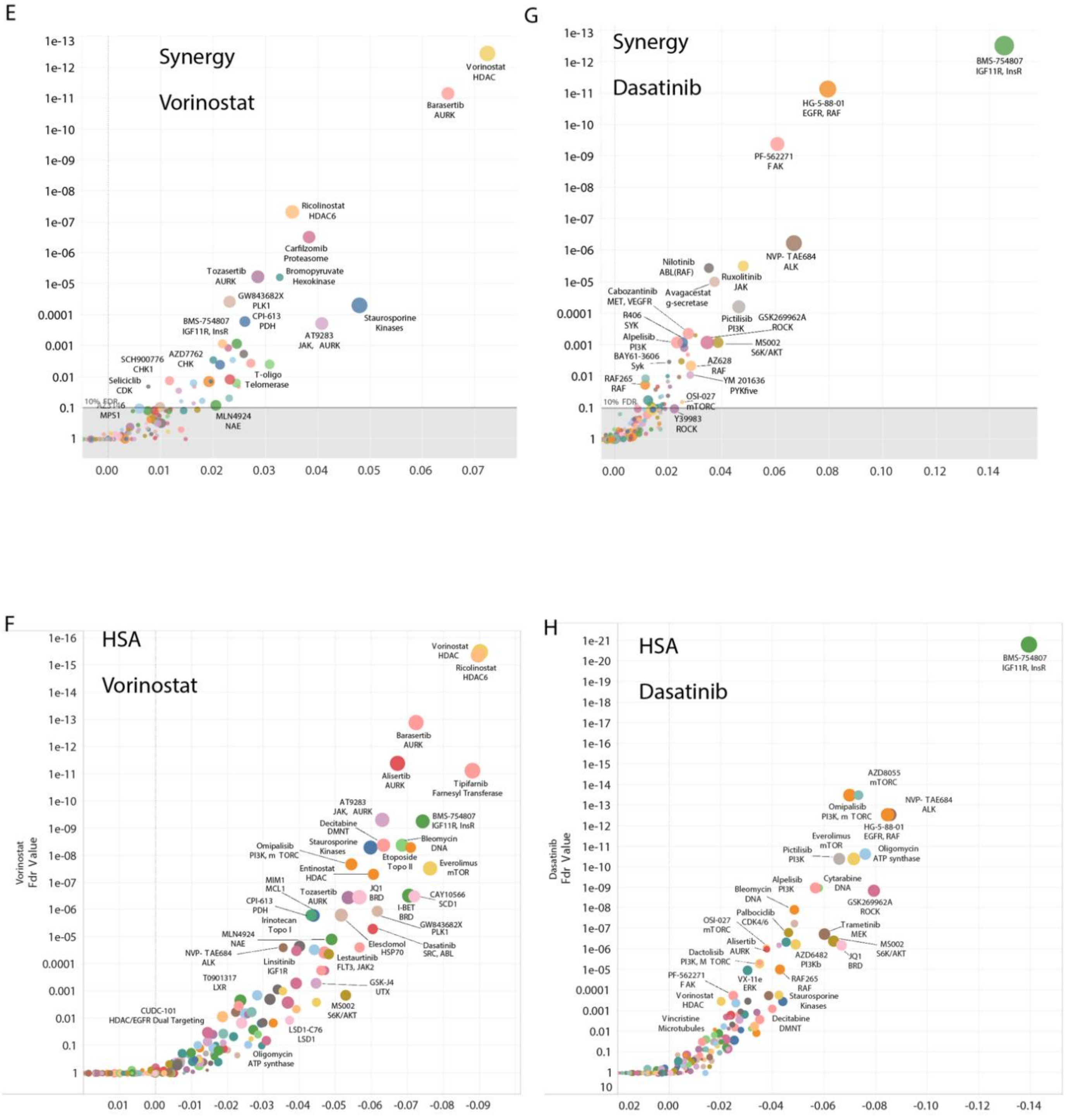

Supplementary Figure 4: Distribution of synergy and HSA scores for selected anchors illustrates global consistency of the two scoring metrics. The impact score for each combination is plotted. X axis: Differential median synergy score across cell lines corresponding to the Log10 ratio of the median synergy (or HSA) score for the indicated library drug over median score of all

other drugs. Y axis: FDR value for statistical enrichment of synergies for the plotted combination over all other tested combinations (with the same anchor). The size of the dots represents the percentile of synergy scores for a given combination falling within the top 5% of all synergy scores for the whole screen (all anchors). Anchors: Pictilisib (pan PI3K), OSI-27 (mTORC), Votinostat (HDAC), Dasatinib (ABL, SRC).

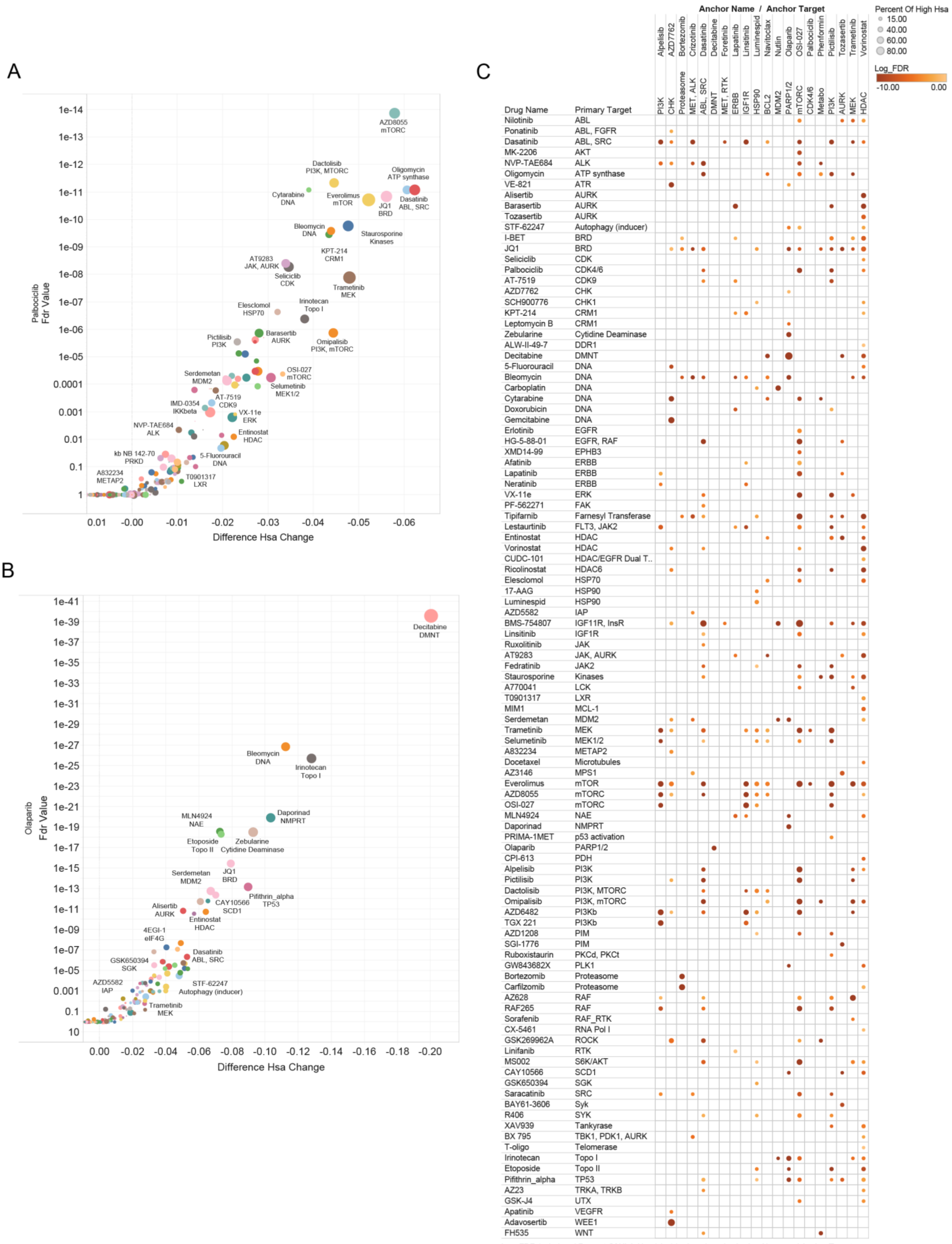
A
Palbociclib
Fdr Value
Difference Hsa Change
1e-14
1e-13
1e-12
1e-11
1e-10
1e-09
1e-08
1e-07
1e-06
1e-05
0.0001
0.001
0.01
0.1
1
0.01
-0.00
-0.01
-0.02
-0.03
-0.04
-0.05
-0.06
AZD8055
mTORC
Dactolisib
PI3K, MTORC
Oligomycin
ATP synthase
Cytarabine
DNA
Everolimus
mTOR
JQ1
BRD
Dasatinib
ABL, SRC
Bleomycin
DNA
Staurosporine
Kinases
KPT-214
CRM1
AT9283
JAK, AURK
Seliciclib
CDK
Trametinib
MEK
Elesclomol
HSP70
Irinotecan
Topo I
Pictilisib
PI3K
Barasertib
AURK
Omipalisib
PI3K, mTORC
Serdemetan
MDM2
OSI-027
mTORC
Selumetinib
MEK1/2
AT-7519
CDK9
IMD-0354
IKKbeta
VX-11e
ERK
NVP-TAE684
ALK
Entinostat
HDAC
kb NB 142-70
PRKD
5-Fluorouracil
DNA
A832234
METAP2
T0901317
LXR
B
Olaparib
Fdr Value
Difference Hsa Change
1e-41
1e-39
1e-37
1e-35
1e-33
1e-31
1e-29
1e-27
1e-25
1e-23
1e-21
1e-19
1e-17
1e-15
1e-13
1e-11
1e-09
1e-07
1e-05
0.001
0.1
10
0.00
-0.02
-0.04
-0.06
-0.08
-0.10
-0.12
-0.14
-0.16
-0.18
-0.20
Decitabine
DMNT
Bleomycin
DNA
Irinotecan
Topo I
MLN4924
NAE
Daporinad
NMPRT
Etoposide
Topo II
Zebularine
Cytidine Deaminase
Serdemetan
MDM2
JQ1
BRD
Pifithrin_alpha
TP53
CAY10566
SCD1
Alisertib
AURK
Entinostat
HDAC
4EGI-1
eIF4G
GSK650394
SGK
Dasatinib
ABL, SRC
AZD5582
IAP
STF-62247
Autophagy (inducer)
Trametinib
MEK
C
Anchor Name / Anchor Target
Alpelisib PI3K
AZD7762 CHK
Bortezomib Proteasome
Crizotinib MET, ALK
Dasatinib ABL, SRC
Decitabine DMNT
Foretinib MET, RTK
Lapatinib ERBB
Linsitinib IGF1R
Luminespid HSP90
Navitoclax BCL2
Nutlin MDM2
Olaparib PARP1/2
OSI-027 mTORC
Palbociclib CDK4/6
Phenformin Metabo
Pictilisib PI3K
Tozasertib AURK
Trametinib MEK
Vorinostat HDAC
Percent Of High Hsa
15.00
40.00
60.00
80.00
Log_FDR
-10.00
0.00
Drug Name Primary Target
Nilotinib ABL
Ponatinib ABL, FGFR
Dasatinib ABL, SRC
MK-2206 AKT
NVP-TAE684 ALK
Oligomycin ATP synthase
VE-821 ATR
Alisertib AURK
Barasertib AURK
Tozasertib AURK
STF-62247 Autophagy (inducer)
I-BET BRD
JQ1 BRD
Seliciclib CDK
Palbociclib CDK4/6
AT-7519 CDK9
AZD7762 CHK
SCH900776 CHK1
KPT-214 CRM1
Leptomycin B CRM1
Zebularine Cytidine Deaminase
ALW-II-49-7 DDR1
Decitabine DMNT
5-Fluorouracil DNA
Bleomycin DNA
Carboplatin DNA
Cytarabine DNA
Doxorubicin DNA
Gemcitabine DNA
Erlotinib EGFR
HG-5-88-01 EGFR, RAF
XMD14-99 EPHB3
Afatinib ERBB
Lapatinib ERBB
Neratinib ERBB
VX-11e ERK
PF-562271 FAK
Tipifarnib Farnesyl Transferase
Lestaurtinib FLT3, JAK2
Entinostat HDAC
Vorinostat HDAC
CUDC-101 HDAC/EGFR Dual T..
Ricolinostat HDAC6
Elesclomol HSP70
17-AAG HSP90
Luminespid HSP90
AZD5582 IAP
BMS-754807 IGF11R, InsR
Linsitinib IGF1R
Ruxolitinib JAK
AT9283 JAK, AURK
Fedratinib JAK2
Staurosporine Kinases
A770041 LCK
T0901317 LXR
MIM1 MCL-1
Serdemetan MDM2
Trametinib MEK
Selumetinib MEK1/2
A832234 METAP2
Docetaxel Microtubules
AZ3146 MPS1
Everolimus mTOR
AZD8055 mTORC
OSI-027 mTORC
MLN4924 NAE
Daporinad NMPRT
PRIMA-1MET p53 activation
Olaparib PARP1/2
CPI-613 PDH
Alpelisib PI3K
Pictilisib PI3K
Dactolisib PI3K, MTORC
Omipalisib PI3K, mTORC
AZD6482 PI3Kb
TGX 221 PI3Kb
AZD1208 PIM
SGI-1776 PIM
Ruboxistaurin PKCd, PKCt
GW843682X PLK1
Bortezomib Proteasome
Carfilzomib Proteasome
AZ628 RAF
RAF265 RAF
Sorafenib RAF_RTK
CX-5461 RNA Pol I
GSK269962A ROCK
Linifanib RTK
MS002 S6K/AKT
CAY10566 SCD1
GSK650394 SGK
Saracatinib SRC
BAY61-3606 Syk
R406 SYK
XAV939 Tankyrase
BX 795 TBK1, PDK1, AURK
T-oligo Telomerase
Irinotecan Topo I
Etoposide Topo II
Pifithrin_alpha TP53
AZ23 TRKA, TRKB
GSK-J4 UTX
Apatinib VEGFR
Adavosertib WEE1
FH535 WNT

Supplementary Figure 5: HSA Scores for selected drugs and overall HSA score matrix. A and B, HSA impact scores for the anchors palbociclib (CDK4/6) and Olaparib (PARP). C: Overview of HSA scores across tested combination. Combinations yielding HSA in at least 15% of the cell lines tested are shown. The size of the dot corresponds to the percentile of cell lines with HSA and the shade to the statistical enrichment of HSA events for the indicated combination over all other tested combinations (darker corresponds to lower FDR).

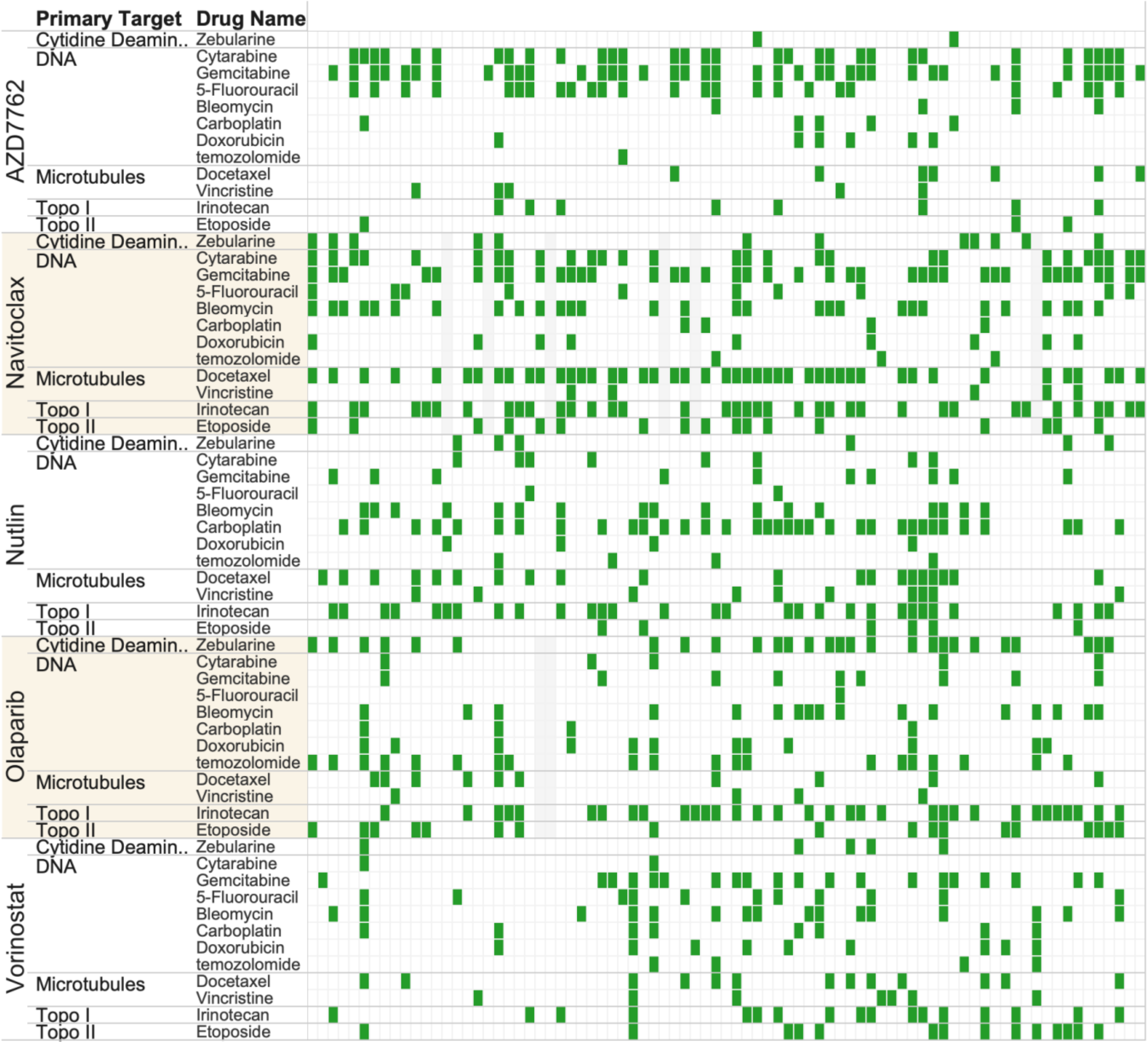

Supplementary Figure 6: Pattern of synergy score for cytotoxic drugs. For each of the indicated anchor drugs (vertical labels) the pattern of synergy for a given library drug (horizontal label) is shown by a color box when the chosen threshold of 0.8 synergy score is passed. Cell lines are in same order across all rows. Gray shading corresponds to missing data.

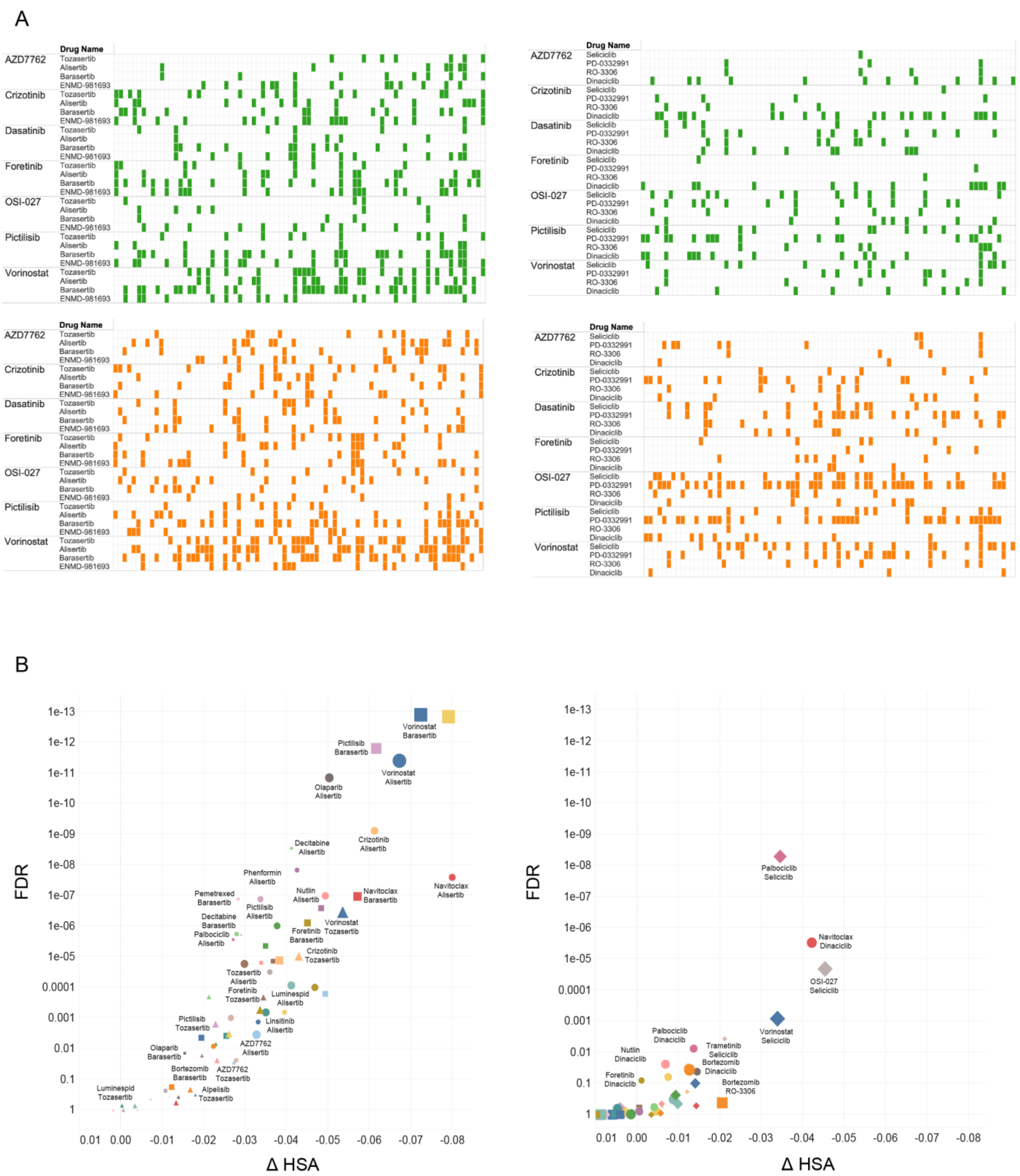

Supplementary Figure 7: Aurora kinase inhibitors are engaged more frequently in synergistic and HSA events than CDK inhibitors. A, Pattern of synergy and HSA events across

selected anchors for the library drugs targeting Aurora Kinases and CDKs. B, Impact score graphs for Aurora Kinase and CDK inhibitors.

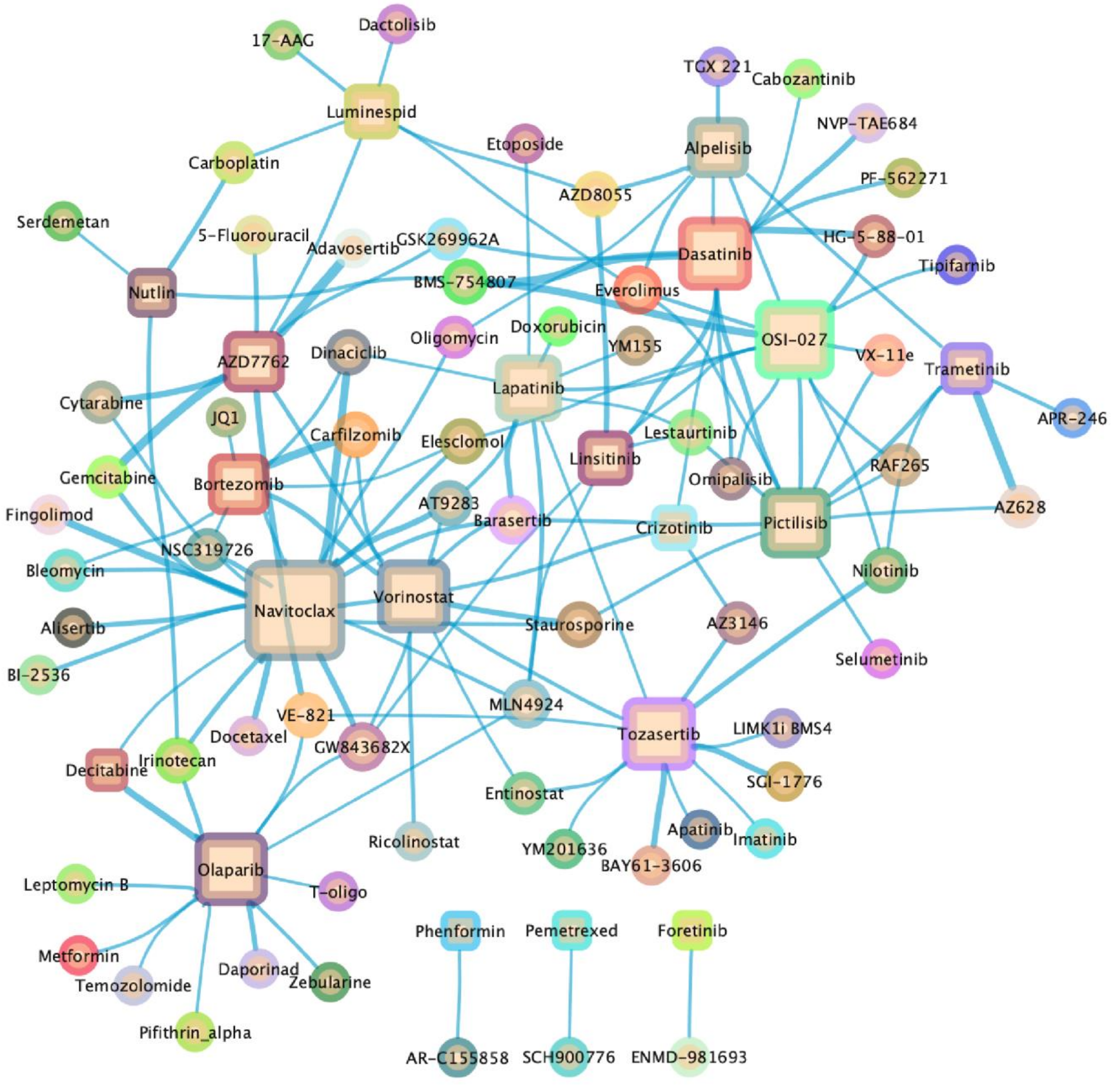

Supplementary Figure 8: Network representation of drug-drug interactions for top 5% synergistic events across all drugs and anchors. Anchor drugs are represented as squares and library drugs as circles. The size of the anchor nodes is proportional to the number of synergistic

events the corresponding drug is involved in. The thickness of the edge between two drugs corresponds to the median value of the synergy scores across all cell lines.

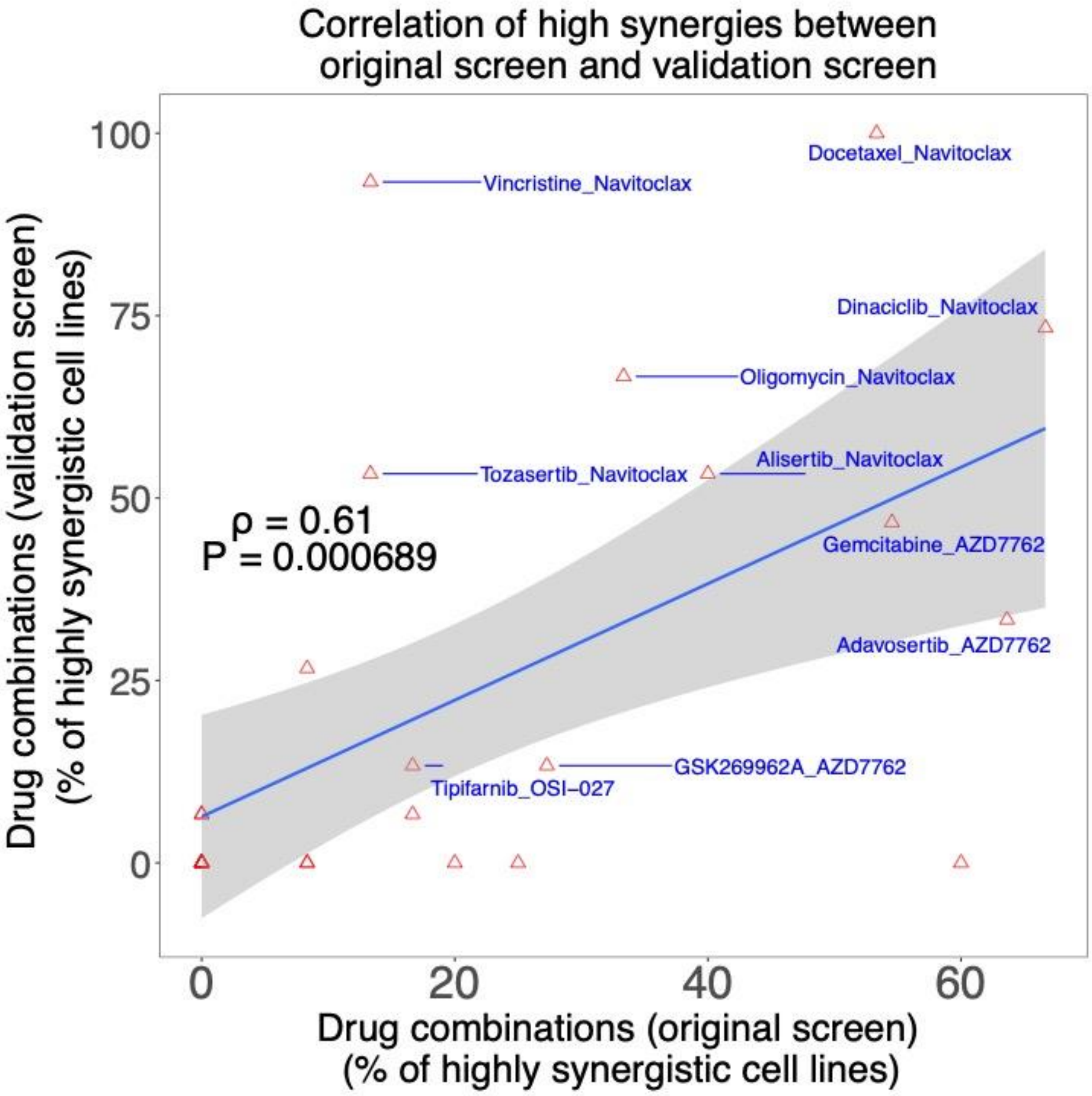

Supplementary figure 9: Validation of high synergistic drug combinations between the original and validation screens. Each drug combination is ranked based on the percentage of cell lines in which they are highly synergistic (Methods), in the original and in the validation screens. Spearman's correlation and p-value between the rankings of the drug combinations between the original and validation screens are shown. Overall, 27 common drug combinations were tested in both screens. Only some drug combination names are displayed for the sake of clarity.

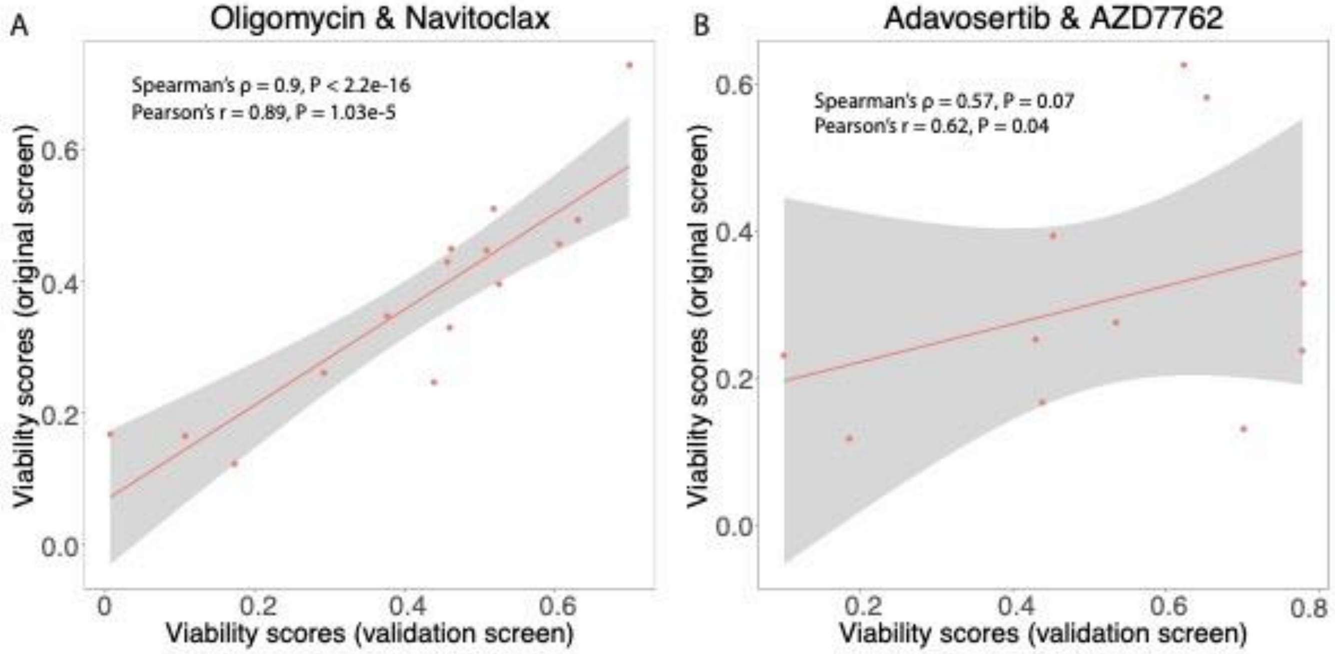

Supplementary figure 10: Correlation between the viability scores of two drug combinations between the original screen and validation screen across all 15 NSCLC cell lines. (A) Oligomycin and Navitoclax; (b) Adavosertib and AZD7762. The correlation values for all 29 drug combinations are shown in Supp. Data S10A. Spearman and pearson correlations and p-values are shown. When P was reported as 0 by the software, it is written as $P < 2.2e-16$.

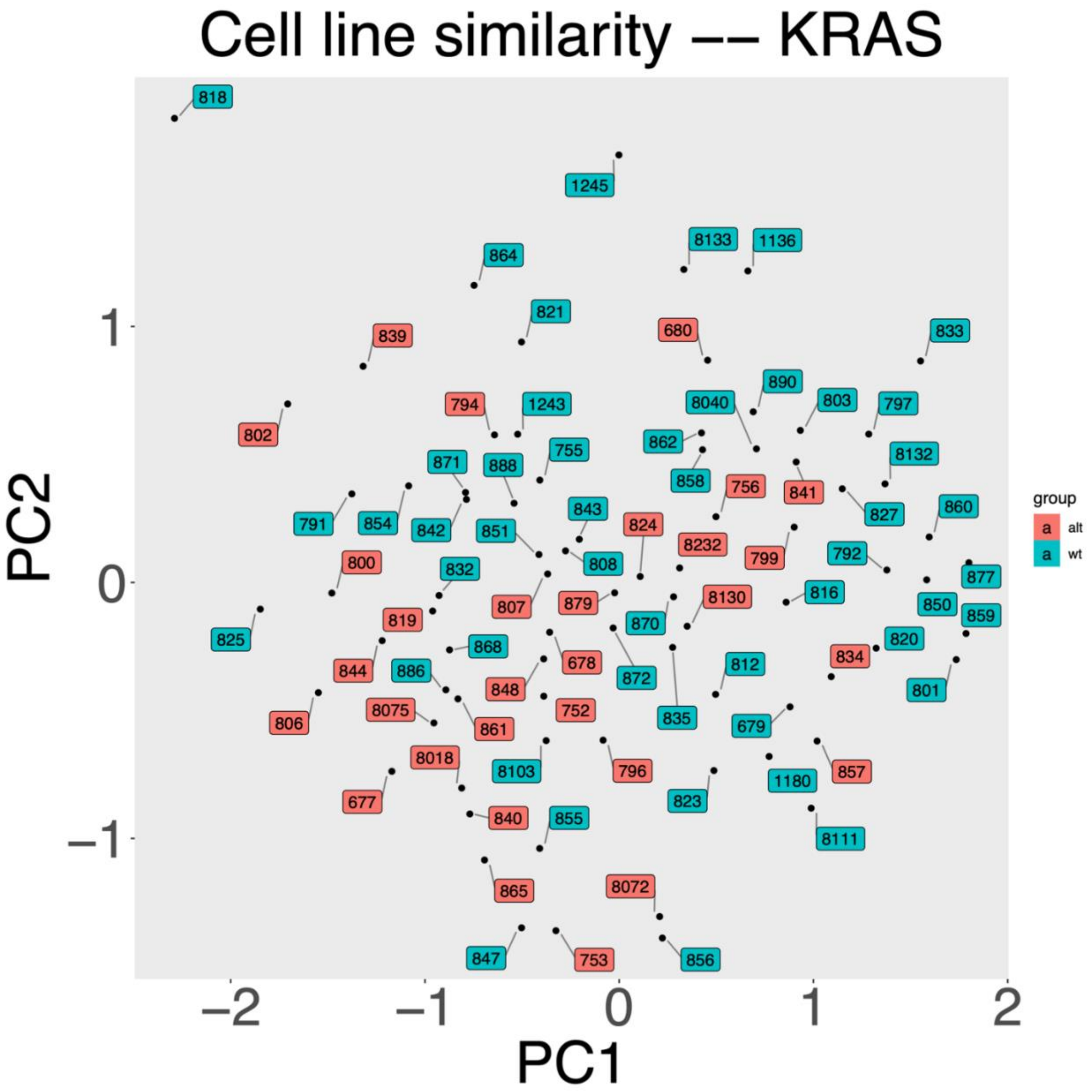

Supplementary Figure 11: Analysis of synergy patterns across drugs for each cell line does not differentiate KRAS WT from KRAS mutant cell lines. Principle component analysis was used to analyze the proximity of cell lines based on the pattern of synergistic events across all anchors and library drugs. Cell lines are color coded based on KRAS mutational status.

Supplementary References

1 Goldoni, M. & Johansson, C. A mathematical approach to study combined effects of toxicants in vitro: evaluation of the Bliss independence criterion and the Loewe additivity model. *Toxicol In Vitro* **21**, 759-769 (2007). https://doi.org:10.1016/j.tiv.2007.03.003

2 Chandu, D. P. Improved greedy algorithm for set covering problem. *arXiv preprint arXiv:1506.04220* (2015).

3 Chvatal, V. A greedy heuristic for the set-covering problem. *Mathematics of operations research* **4**, 233-235 (1979).

4 Grossman, T., ; Wool, A. . Computational experience with approximation algorithms for the set covering problem. *European journal of operational research* **101**, 81-92 (1997).

5 Bajrami, I. *et al.* Synthetic lethality of PARP and NAMPT inhibition in triple-negative breast cancer cells. *EMBO Mol Med* **4**, 1087-1096 (2012). https://doi.org:10.1002/emmm.201201250

6 Heske, C. M. *et al.* Matrix Screen Identifies Synergistic Combination of PARP Inhibitors and Nicotinamide Phosphoribosyltransferase (NAMPT) Inhibitors in Ewing Sarcoma. *Clin Cancer Res* **23**, 7301-7311 (2017). https://doi.org:10.1158/1078-0432.CCR-17-1121

7 Harrington, L. S., Findlay, G. M. & Lamb, R. F. Restraining PI3K: mTOR signalling goes back to the membrane. *Trends Biochem Sci* **30**, 35-42 (2005). https://doi.org:10.1016/j.tibs.2004.11.003

8 Yoneyama, Y. *et al.* Serine Phosphorylation by mTORC1 Promotes IRS-1 Degradation through SCFbeta-TRCP E3 Ubiquitin Ligase. *iScience* **5**, 1-18 (2018). https://doi.org:10.1016/j.isci.2018.06.006

9 Kovalski, J. R., Shanderson, R. L. & Khavari, P. A. Ras functional proximity proteomics establishes mTORC2 as new direct ras effector. *Oncotarget* **10**, 5126-5135 (2019). https://doi.org:10.18632/oncotarget.27025

10 Asghar, U., Witkiewicz, A. K., Turner, N. C. & Knudsen, E. S. The history and future of targeting cyclin-dependent kinases in cancer therapy. *Nat Rev Drug Discov* **14**, 130-146 (2015). https://doi.org:10.1038/nrd4504

11 Fry, D. W. *et al.* Specific inhibition of cyclin-dependent kinase 4/6 by PD 0332991 and associated antitumor activity in human tumor xenografts. *Mol Cancer Ther* **3**, 1427-1438 (2004).

12 Cristofanilli, M. *et al.* Fulvestrant plus palbociclib versus fulvestrant plus placebo for treatment of hormone-receptor-positive, HER2-negative metastatic breast cancer that progressed on previous endocrine therapy (PALOMA-3): final analysis of the multicentre, double-blind, phase 3 randomised controlled trial. *Lancet Oncol* **17**, 425-439 (2016). https://doi.org:10.1016/S1470-2045(15)00613-0

13 Lim, J. S., Turner, N. C. & Yap, T. A. CDK4/6 Inhibitors: Promising Opportunities beyond Breast Cancer. *Cancer Discov* **6**, 697-699 (2016). https://doi.org:10.1158/2159-8290.CD-16-0563

14 Puyol, M. *et al.* A synthetic lethal interaction between K-Ras oncogenes and Cdk4 unveils a therapeutic strategy for non-small cell lung carcinoma. *Cancer Cell* **18**, 63-73 (2010). https://doi.org:10.1016/j.ccr.2010.05.025

15 Patnaik, A. *et al.* Efficacy and Safety of Abemaciclib, an Inhibitor of CDK4 and CDK6, for Patients with Breast Cancer, Non-Small Cell Lung Cancer, and Other Solid Tumors. *Cancer Discov* **6**, 740-753 (2016). https://doi.org:10.1158/2159-8290.CD-16-0095
16 Pacheco, J. & Schenk, E. CDK4/6 inhibition alone and in combination for non-small cell lung cancer. *Oncotarget* **10**, 618-619 (2019). https://doi.org:10.18632/oncotarget.26545
17 Xue, Y. *et al.* SMARCA4 loss is synthetic lethal with CDK4/6 inhibition in non-small cell lung cancer. *Nat Commun* **10**, 557 (2019). https://doi.org:10.1038/s41467-019-08380-1
18 Wander, S. A. *et al.* The Genomic Landscape of Intrinsic and Acquired Resistance to Cyclin-Dependent Kinase 4/6 Inhibitors in Patients with Hormone Receptor-Positive Metastatic Breast Cancer. *Cancer Discov* **10**, 1174-1193 (2020). https://doi.org:10.1158/2159-8290.CD-19-1390
19 Hafner, M. *et al.* Multiomics Profiling Establishes the Polypharmacology of FDA-Approved CDK4/6 Inhibitors and the Potential for Differential Clinical Activity. *Cell Chem Biol* **26**, 1067-1080 e1068 (2019). https://doi.org:10.1016/j.chembiol.2019.05.005
20 Thomas, S. M. & Brugge, J. S. Cellular functions regulated by Src family kinases. *Annu Rev Cell Dev Biol* **13**, 513-609 (1997). https://doi.org:10.1146/annurev.cellbio.13.1.513
21 Begley, M. J. *et al.* EGF-receptor specificity for phosphotyrosine-primed substrates provides signal integration with Src. *Nat Struct Mol Biol* **22**, 983-990 (2015). https://doi.org:10.1038/nsmb.3117
22 Jabbour, E. & Kantarjian, H. Chronic myeloid leukemia: 2020 update on diagnosis, therapy and monitoring. *Am J Hematol* **95**, 691-709 (2020). https://doi.org:10.1002/ajh.25792
23 Galkin, A. V. *et al.* Identification of NVP-TAE684, a potent, selective, and efficacious inhibitor of NPM-ALK. *Proc Natl Acad Sci U S A* **104**, 270-275 (2007). https://doi.org:10.1073/pnas.0609412103
24 Seashore-Ludlow, B. *et al.* Harnessing Connectivity in a Large-Scale Small-Molecule Sensitivity Dataset. *Cancer Discov* **5**, 1210-1223 (2015). https://doi.org:10.1158/2159-8290.CD-15-0235
25 Zullo, K. M. *et al.* Aurora A Kinase Inhibition Selectively Synergizes with Histone Deacetylase Inhibitor through Cytokinesis Failure in T-cell Lymphoma. *Clin Cancer Res* **21**, 4097-4109 (2015). https://doi.org:10.1158/1078-0432.CCR-15-0033
26 Bertolin, G. *et al.* Aurora kinase A localises to mitochondria to control organelle dynamics and energy production. *Elife* **7** (2018). https://doi.org:10.7554/eLife.38111
27 Bertolin, G. & Tramier, M. Insights into the non-mitotic functions of Aurora kinase A: more than just cell division. *Cell Mol Life Sci* **77**, 1031-1047 (2020). https://doi.org:10.1007/s00018-019-03310-2
28 Otto, T. *et al.* Stabilization of N-Myc is a critical function of Aurora A in human neuroblastoma. *Cancer Cell* **15**, 67-78 (2009). https://doi.org:10.1016/j.ccr.2008.12.005
29 Crystal, A. S. *et al.* Patient-derived models of acquired resistance can identify effective drug combinations for cancer. *Science* **346**, 1480-1486 (2014). https://doi.org:10.1126/science.1254721
30 Donnella, H. J. *et al.* Kinome rewiring reveals AURKA limits PI3K-pathway inhibitor efficacy in breast cancer. *Nat Chem Biol* **14**, 768-777 (2018). https://doi.org:10.1038/s41589-018-0081-9

31 Shah, K. N. *et al.* Aurora kinase A drives the evolution of resistance to third-generation EGFR inhibitors in lung cancer. *Nat Med* **25**, 111-118 (2019). https://doi.org:10.1038/s41591-018-0264-7

32 Friedman, A. A. *et al.* Landscape of Targeted Anti-Cancer Drug Synergies in Melanoma Identifies a Novel BRAF-VEGFR/PDGFR Combination Treatment. *Plos One* **10**, e0140310 (2015). https://doi.org:10.1371/journal.pone.0140310

33 Guieze, R. *et al.* Mitochondrial Reprogramming Underlies Resistance to BCL-2 Inhibition in Lymphoid Malignancies. *Cancer Cell* **36**, 369-384 e313 (2019). https://doi.org:10.1016/j.ccell.2019.08.005

34 Gross, A. & Katz, S. G. Non-apoptotic functions of BCL-2 family proteins. *Cell Death Differ* **24**, 1348-1358 (2017). https://doi.org:10.1038/cdd.2017.22

35 Gao, H. *et al.* High-throughput screening using patient-derived tumor xenografts to predict clinical trial drug response. *Nat Med* **21**, 1318-1325 (2015). https://doi.org:10.1038/nm.3954
