# Supplementary material for: A landscape of response to drug combinations in non-small cell lung cancer
Source: Nat Commun. 2023 Jun 28;14:3830. doi: 10.1038/s41467-023-39528-9 (PMC10307832; doi:10.1038/s41467-023-39528-9)
Supplement: Supplementary file 3 — Reporting Summary [file 41467_2023_39528_MOESM3_ESM.pdf]

nature portfolio

Corresponding author(s): Craig Thomas, Eytan Ruppin, Cyril Benes

Last updated by author(s): Apr 12, 2023

Reporting Summary

Nature Portfolio wishes to improve the reproducibility of the work that we publish. This form provides structure for consistency and transparency in reporting. For further information on Nature Portfolio policies, see our Editorial Policies and the Editorial Policy Checklist.

Statistics

For all statistical analyses, confirm that the following items are present in the figure legend, table legend, main text, or Methods section.

n/a	Confirmed	
☐	☒	The exact sample size (*n*) for each experimental group/condition, given as a discrete number and unit of measurement
☐	☒	A statement on whether measurements were taken from distinct samples or whether the same sample was measured repeatedly
☐	☒	The statistical test(s) used AND whether they are one- or two-sided<br>*Only common tests should be described solely by name; describe more complex techniques in the Methods section.*
☐	☒	A description of all covariates tested
☐	☒	A description of any assumptions or corrections, such as tests of normality and adjustment for multiple comparisons
☐	☒	A full description of the statistical parameters including central tendency (e.g. means) or other basic estimates (e.g. regression coefficient) AND variation (e.g. standard deviation) or associated estimates of uncertainty (e.g. confidence intervals)
☐	☒	For null hypothesis testing, the test statistic (e.g. *F*, *t*, *r*) with confidence intervals, effect sizes, degrees of freedom and *P* value noted<br>*Give P values as exact values whenever suitable.*
☒	☐	For Bayesian analysis, information on the choice of priors and Markov chain Monte Carlo settings
☐	☒	For hierarchical and complex designs, identification of the appropriate level for tests and full reporting of outcomes
☐	☒	Estimates of effect sizes (e.g. Cohen's *d*, Pearson's *r*), indicating how they were calculated

Our web collection on statistics for biologists contains articles on many of the points above.

Software and code

Policy information about availability of computer code

Data collection	R software was used for this analysis.
Data analysis	Code has been made available.

For manuscripts utilizing custom algorithms or software that are central to the research but not yet described in published literature, software must be made available to editors and reviewers. We strongly encourage code deposition in a community repository (e.g. GitHub). See the Nature Portfolio guidelines for submitting code & software for further information.

Data

Policy information about availability of data

All manuscripts must include a data availability statement. This statement should provide the following information, where applicable:

- Accession codes, unique identifiers, or web links for publicly available datasets
- A description of any restrictions on data availability
- For clinical datasets or third party data, please ensure that the statement adheres to our policy

The processed drug combination screen data is provided in Tables S1, S2, S8. The raw original screen data is available here:
https://hpc.nih.gov/~Lab_ruppin/raw_data_for_NSCLC_paper.zip
Source data are provided with this paper. Protein-protein interaction network scores from the publicly available STRING database (downloaded on Aug. 8, 2019) was used.

Human research participants

Policy information about studies involving human research participants and Sex and Gender in Research.

Reporting on sex and gender	Not applicable
Population characteristics	Not applicable
Recruitment	Not applicable
Ethics oversight	Not applicable

Note that full information on the approval of the study protocol must also be provided in the manuscript.

Field-specific reporting

Please select the one below that is the best fit for your research. If you are not sure, read the appropriate sections before making your selection.

☒ Life sciences ☐ Behavioural & social sciences ☐ Ecological, evolutionary & environmental sciences

For a reference copy of the document with all sections, see nature.com/documents/nr-reporting-summary-flat.pdf

Life sciences study design

All studies must disclose on these points even when the disclosure is negative.

Sample size	Drug combinations with 21 anchor drugs (1 dose) and 242 library drugs (5 doses). Tested on 81 cell lines. This is one of the largest drug combination datasets available. The sample size was chosen to capture the diversity of genetic and transciptional profiles in non-small cell lung cancer. The methodologies and type of data reported here do not require pre-determined sample size.
Data exclusions	No data excluded
Replication	Screening was performed using two technical replicates (2 wells with identical treatment for all conditions tested). A coefficient of variation of less than 25% was set as a quality control pass threshold. Data corresponding to plates not passing the quality control were not used and experiments were repeated to re-acquire these data.
Randomization	Randomization is not an approach used in the type of studies reported here, the type of data collected do not allow for randomization to improve robustness of conclusions.
Blinding	None of the type of analyses conducted in this study require blinding as the analyses are based on statistical methods from unselected experimental data.

Reporting for specific materials, systems and methods

We require information from authors about some types of materials, experimental systems and methods used in many studies. Here, indicate whether each material, system or method listed is relevant to your study. If you are not sure if a list item applies to your research, read the appropriate section before selecting a response.

Materials & experimental systems

n/a	Involved in the study	
☒	☐	Antibodies
☐	☒	Eukaryotic cell lines
☒	☐	Palaeontology and archaeology
☒	☐	Animals and other organisms
☒	☐	Clinical data
☒	☐	Dual use research of concern

Methods

n/a	Involved in the study	
☒	☐	ChIP-seq
☒	☐	Flow cytometry
☒	☐	MRI-based neuroimaging

Eukaryotic cell lines

Policy information about cell lines and Sex and Gender in Research

Cell line source(s)	Source:<br>A-427:ATCC, A549:ATCC, Calu-6:ATCC, ChaGo-K-1:ATCC, NCI-H1299:ATCC, NCI-H1355:ATCC, NCI-H1395:ATCC, NCI-H1437:ATCC, NCI-H1563:ATCC, NCI-H1623:ATCC, NCI-H1648:ATCC, NCI-H1650:ATCC, NCI-H1651:ATCC, NCI-H1666:ATCC, NCI-H1703:ATCC, NCI-H1734:ATCC, NCI-H1755:ATCC, NCI-H1792:ATCC, NCI-H1793:ATCC, NCI-H1915:ATCC, NCI-H1944:ATCC, NCI-H1975:ATCC, NCI-H1993:ATCC, NCI-H2009:ATCC, NCI-H2023:ATCC, NCI-H2085:ATCC, NCI-H2087:ATCC, NCI-H2126:ATCC, NCI-H2170:ATCC, NCI-H2228:ATCC, NCI-H23:ATCC, NCI-H2342:ATCC, NCI-H2347:ATCC, NCI-H2405:ATCC, NCI-H358:ATCC, NCI-H441:ATCC, NCI-H460:ATCC, NCI-H520:ATCC, NCI-H522:ATCC, NCI-H596:ATCC, NCI-H647:ATCC, NCI-H650:ATCC, NCI-H661:ATCC, NCI-H727:ATCC, NCI-H838:ATCC, SK-MES-1:ATCC, SW 1573:ATCC, SW 900:ATCC, UMC-11:ATCC, NCI-H3122:DFCI, BEN:DSMZ, CAL-12T:DSMZ, EPLC-272H:DSMZ, HCC-15:DSMZ, HCC-366:DSMZ, HCC-44:DSMZ, HCC-78:DSMZ, HCC-827:DSMZ, LCLC-103H:DSMZ, LCLC-97TM1:DSMZ, LOU-NH91:DSMZ, LXF-289:DSMZ, COR-L 105:ECACC, CO-L23:ECACC, NCI-H322M:NCI, PC-14:ECACC, SK-LU-1:ECACC, ABC-1:JHSF, EBC-1:JHSF, HARA:JHSF, LK-2:JHSF, LU99A:JHSF, PC-3:JHSF, RERF-LC-KJ:JHSF, EKVX:NCI, H3255:NCI, HOP-62:NCI, IA-LM:RIKEN, LC-2-ad:RIKEN, EMC-BAC-2: WTSI, A201T:UPMC.
Authentication	Stocks were made from commercial source or original source to allow for use of cells from stock vials within 10 passages. SNP analysis was used to check for cross-contamination of stocks as reported in Garnett et al. Nature 2012.
Mycoplasma contamination	All cell lines were tested for mycoplasma contamination before drug screening and only Mycoplasma free cells were used.
Commonly misidentified lines<br>(See ICLAC register)	No commonly misidentified cell lines were used in the study.
