# Supplementary material for: A landscape of response to drug combinations in non-small cell lung cancer
Source: Nat Commun. 2023 Jun 28;14:3830. doi: 10.1038/s41467-023-39528-9 (PMC10307832; doi:10.1038/s41467-023-39528-9)
Supplement: Supplementary file 4 — Description of Additional Supplementary Files [file 41467_2023_39528_MOESM4_ESM.docx]

**Description of Additional Supplementary Files**

Supplementary Data 1

Description:

| a: Anchor Drug names and their main targets |
| --- |
| b: Library Drug names and their main targets |
| c: Anchor drugs, concentrations used, and the complete list of targets for each drug. |
| d: Library drugs, concentrations used, and the complete list of targets for each drug. |
| e: Cell ID and Cell Line name |
| f: Mutational/alteration information and histology for non-small-cell lung cancer (NSCLC) cell lines for different driver genes. |

Supplementary Data 2

Description: Processed data from the drug combination *in vitro* screen

| a: Processed drug response dataset from *in vitro* screen |
| --- |
| b: Processed drug response dataset from *in vitro* screen that was used in the analysis of this manuscript. |
| c: Ranking of drug combinations based on the percentage of cell lines where they are highly synergistic (threshold of high synergy is based on top 5 percentile). |

Supplementary Data 3

Description:

| a: Minimum number of library drugs for each anchor drug to cover a certain percentage of cell lines with high synergies. |
| --- |
| b: All drug combinations ranked based on their super-sensitive effect. Percent of cell lines with super sensitive effect for each combination is shown. |
| c: Top drug combinations ranked based on their super-sensitive effect. Percent of cell lines with super sensitive effect for each combination is shown. |
| d: Drug combinations which is highly effective (viability less than 0.4) but single drug is not effective (viability greater than 0.75) is shown. Only combinations which show this effect in at least 4 percent of the cell lines are shown. |

Supplementary Data 4

Description:

| a: Drug combinations more effective (less viability) in KRAS mutant compared to wild-type cell lines. One-sided Wilcoxon rank-sum p-values shown. |
| --- |
| b: Drug combinations more effective (less viability) in EGFR mutant compared to wild-type cell lines. One-sided Wilcoxon rank-sum p-values shown. |
| c: Drug combinations more effective (less viability) in PIK3CA mutant compared to wild-type cell lines. One-sided Wilcoxon rank-sum p-values shown. |
| d: Drug combinations less effective (more viability) in TP53 mutant compared to wild-type cell lines. One-sided Wilcoxon rank-sum p-values shown. |
| e: Anchor drugs with an imbalance in synergy number (high synergies) favoring STK11 wild-type compared to STK11 mutants, among KRAS wild-type cell lines (i.e STK11 wild-type + KRAS wild-type in comparison with STK11 mutant + KRAS wild-type). |
| f: Drug combinations less effective (more viability) in TERT mutant compared to wild-type cell lines. One-sided Wilcoxon rank-sum p-values shown. FDR < 0.2. |
| g: Drug combinations less effective (more viability) in MACF1 mutant compared to wild-type cell lines. One-sided Wilcoxon rank-sum p-values shown. FDR < 0.2. |

Supplementary Data 5

Description:

| a: Drug combinations with improved survival effect than individual drugs. Gene expression based analysis. High synergistic drug combinations where individual drugs have less than 4 targets are considered. |
| --- |
| b: Drug combinations with improved survival effect than individual drugs. Copy-number based analysis. Highly synergistic drug combinations where individual drugs have less than 4 targets are considered. |
| c: Drug combinations with improved survival effect than individual drugs. Gene expression based analysis. All drug combinations where individual drugs have less than 4 targets are considered. |
| d: Drug combinations with improved survival effect than individual drugs. Copy-number based analysis. All drug combinations where individual drugs have less than 4 targets are considered. |

Supplementary Data 6

Description:

| a: Computationally predicted synthetic lethal (SL) gene pairs. ISLE was used on lung cancer TCGA data to predict clinically relevant SL pairs (FDR < 0.2). The p-values of the 4 screens steps are also shown. |
| --- |
| b: All drug combinations were individual drugs have less than 4 targets and which target computational synthetic lethal (SL) pairs. No. of SL pairs targeted by the drug combination is shown. |
| c: Highly synergistic drug combinations were individual drugs have less than 4 targets and which target computational synthetic lethal (SL) pairs. No. of SL pairs targeted by the drug combination is shown. |
| d: Experimentally derived synthetic lethal gene pairs. |
| e: All drug combinations were individual drugs have less than 4 targets and which target experimentally-derived synthetic lethal (SL) pairs. No. of SL pairs targeted by the drug combination is shown. |
| f: Highly synergistic drug combinations were individual drugs have less than 4 targets and which target experimentally-derived synthetic lethal (SL) pairs. No. of SL pairs targeted by the drug combination is shown. |

Supplementary Data 7

Description:

Shows drug combinations with empirical p-values. Empirical p-values were computed for synergy scores for each drug combination at the 5 library drug doses and the second-best synergy score by converting the log-transformed synergy scores (to make the synergy ratios more normal distribution like) into z-scores and then computing empirical p-values assuming a normal distribution. Similarly, empirical p-values were also computed for HSA values for all cell lines and combinations.

Supplementary Data 8

Description: Data for the validation screen.

Supplementary Data 9

Description: Shows spearman's correlation for the synergy scores between the two replicates for each drug combination, across all cell lines and library drug doses.

Supplementary Data 10

Description : Spearman's and Pearson's corre;lation for the viability values of each drug combination across a subset of cell lines between the original and validation screens.

Supplementary Data 11

Description:

a. Drugs which are more effective (less viability) in 15 NSCLC cancer cell lines in comparison to the 2 normal non-cancerous cell lines.
b. Drugs more synergistic in 15 NSCLC cancer cell lines in comparison to the 2 normal non-cancerous cell lines.

Supplementary Data 12

Description: The raw original screen data is made available here.
