# Supplementary material for: A landscape of response to drug combinations in non-small cell lung cancer
Source: Nat Commun. 2023 Jun 28;14:3830. doi: 10.1038/s41467-023-39528-9 (PMC10307832; doi:10.1038/s41467-023-39528-9)
Supplement: Supplementary file 16 — Supplementary Dataset S12 [file 41467_2023_39528_MOESM16_ESM.zip › raw_data_for_NSCLC_paper/for_paper_Combo_NSCLC_ReadMe_OriginalScreen.rtf]

Readme file for original drug combination in vitro screen for non-small-cell lung cancer (NSCLC) done at Massachusetts General Hospital. 


Assay:

Cells are seeded at optimal density determined by prior testing in the assay format.
Drugged plates are seeded at Day 0 ; Drugged at Day 1; Fixed and stained at Day 6 = 5 days in drug.
The cell count is performed by nuclear stain and enumeration using an automated imager and a dedicated software. Count accuracy was verified for each cell line before screening and routinely checked during the screen.


Combination setting: 

Cells are exposed to 242 drugs (“library drugs) either alone or in combination with an “anchor” drug.
There are 21 anchor drugs.

Library drugs are tested at 5 different doses.
The different doses and drugs are randomly assigned across the plate rather than in adjacent wells to minimize artifacts of “regional” difference in viability and to allow for median polish of the data.


Drug doses:
Each library drug is used at 5 different doses listed in the “COMBO_Library.CSV” file
Doses are labelled D1 to D5 and concentration in micro molar listed.
The location of the well is listed as well as the corresponding Zone location.

Some of the anchor drugs were used at different concentrations based on potency observed in initial stages of the screen. These concentrations are listed in the Anchor Drug file.

Experimental Set Up.
1 plate is drugged with single drugs at 5 different concentrations.
1 “sister” plate seeded concomitantly is drugged with the same single drugs + an Anchor drug.
Thus a given plate contains either only single drugs or combinations (+ anchor alone for the wells of the “anchor” plate that correspond to no drug in the single drug plate).
These wells are labeled as CRL in all plates: in the single drug plate (NOT anchor) they are untreated (DMSO) wells. In the Anchor plate these are Anchor alone wells.

In other words library drugs are added to 2 identical plates. As a second step one receives DMSO (control solvent) in all wells the other receives the anchor drug in all wells.


In addition, for each run (a given seed date) 2 replicate plates are used (2 single drug plates and 2 combination plates).

Data and plate zone:
There are 3 “zones” in the 1536 plate that are assigned based on known issues of quality of data due to evaporation problems etc.
Zone 1 corresponds to the 2 most outer wells - Some low priority drugs are in Zone 1. Otherwise, it is used for plate orientation check if necessary (see below). These drugs are not a part of the paper and are not shown.
Zone 2 corresponds to the 2 next inside well series. These should have fairly low noise data 
Zone 3 corresponds to the rest of the wells (the rest most inner wells). This is the top quality data area.

QC:
We are sending you only plates that have passed QC.

All plates need to pass CV =< 25% for the control wells in Zone 3 (CV of the cell count values across the control replicates in a given plate).
All plates should demonstrate proliferation corresponding to at least 1 doubling (see below).
All “anchored” plates need to have a sister DMSO plate that has passed the above QC requirements in the same run.

Proliferation test:

At the same time that cells are seeded in plates that will be drugged a separate pair of plate is seeded to measure proliferation.
One of these plates is fixed and cells counted on “Day 1” = this is the Day after seeding (Day of seeding is Day 0).
The other plate is fixed and cells counted on “Day 6” = 5 days after corresponding to the time that the drugged plates have been drugged.
Drugged plates are seeded at Day 0 ; Drugged at Day 1; Fixed and stained at Day 6 = 5 days in drug.

The cell counts in Day1 plate and day 6 plates matched to the drug plates are provided.
Proliferation in the drugged plate can be accounted by comparing Day 1 plate counts to Single Drug plate Control well counts  (These wells are in the drugged plate and received DMSO).
This should be close to the D1 D6 plate comparison but because of plate handling differences it might be different and the most accurate proliferation rate is derived from D1/Single agent plate comparison. D1 / D6 pairs are useful to detect potential issues of seeding in the drugged plates - mismatched (technical errors) etc.


Control Wells:
A number of control wells are in each plate CRL wells are either No drug wells or anchor alone wells as explained above.
The SE1 - SE13 are wells at the periphery of the plate that are used to verify the orientation of the plate during drugging if necessary. They are also used to get an estimate of whether drug effect can be registered on each plate (although routinely we use the D1- D5 wells of the Staurosporin drug Cpd 307 that are located off the periphery). The wells contain Staurosporin a pan killing agent (hen used at high dose). This compound is Cpd #999 in this “control” set-up with 13 different doses used for this control setting.
These are SE1 to SE13 doses.

Note: Staurosporin in also used as a library drug (Cpd #307) with the usual 5 doses.


Files: 
General rules: 
Barcodes are unique = one plate
Cell IDs are unique = one cell line
Drug IDs are unique = one drug (compound).

Included Files:

“for_paper_COMBO_Library.csv” : Library drug IDs, concentration (in micro molar) and position in the plate.


“Supp_Table_S1_Drug_CellLine_Details.xlsx” : 
IDs, Name, Alternate name, doses for library drugs.
Cell lines unique IDs. Name and characetristics.
The Cell ID is the same one used throughout our dataset and can be used to assign genomic data to each cell line.
List of anchor drugs ; version and concentration. (These are Drug #2 combined with the library drugs see below).

“ComboD1D6RawData_20140708-20160211.txt”: Proliferation dataset. 
Barcode	Replicate	Day	SeedDate	CellID	CellsPerWell	PlatedDensity	CellCount	
Seed Date can be used to match the proliferation test plates in this file with the Drugged plates (see below).
CellsPer Well is the intended seeded number at Day0 - CellCount is the actual cell count out of the imager.
Cell count is the number of cells in a well - central wells are counted for these plates.
PlatedDensity refers to the cell suspension used to seed (same information as CellsPerWell).
Replicate: The replicate ID. Either 1 or 2 for the 2 plates seeded concomitantly.
CellID; the unique ID of the cell line
Day; The corresponding Read Day = Either 1 or 6 = Plate read at Day1 or at Day 6 - For a given cell line / Date / Density These are different plates seeded together (different barcode)


“for_paper_ComboRawData_20140708-20160211_zone3_zone2.txt”: The drugged plates data. Raw counts of cells in each wells.
To match to the proliferation plate uniquely one needs to use SeedDate & Cell ID & Plated Density 
In most cases there is only 1 plated density for a given date and cell ID but there are exceptions.
“Grp”: Ignore this it is the same as the “Dose” column.

AnchorID = Unique Drug ID
Anchor Ver = Anchor version (concentration see Anchor table)
Well ID: position of the well on the plate
Compound” Drug ID
Concentration: The concentration (final) of the Library drug in micro molar
Dose: The Dose ID
Zone: the Zone (region) of the plate
Cell Count: the actual cell count from the imager.


SeedDate	Barcode	CellID	AnchorID	AnchorVer	PlatedDensity	WellID	Grp	Compound	Concentration	Dose	Zone	CellCount	
